# Efficacy of interventions and techniques on adherence to physiotherapy in adults: an overview of systematic reviews and panoramic meta-analysis

**DOI:** 10.1186/s13643-024-02538-9

**Published:** 2024-05-21

**Authors:** Clemens Ley, Peter Putz

**Affiliations:** 1 Department Health Sciences, Physiotherapy, FH Campus Wien University of Applied Sciences, Favoritenstrasse 226, 1100 Vienna, Austria; 2 Department Health Sciences, Competence Center INDICATION, FH Campus Wien, University of Applied Sciences, Favoritenstrasse 226, 1100 Vienna, Austria

**Keywords:** Umbrella review, Physical therapy, Exercise, Rehabilitation, Adherence, Compliance, Motivation, Behaviour change techniques, Effectiveness, Efficacy

## Abstract

**Background:**

Adherence to physiotherapeutic treatment and recommendations is crucial to achieving planned goals and desired health outcomes. This overview of systematic reviews synthesises the wide range of additional interventions and behaviour change techniques used in physiotherapy, exercise therapy and physical therapy to promote adherence and summarises the evidence of their efficacy.

**Methods:**

Seven databases (PEDro, PubMed, Cochrane Library, Web of Science, Scopus, PsycINFO and CINAHL) were systematically searched with terms related to physiotherapy, motivation, behaviour change, adherence and efficacy (last searched on January 31, 2023). Only systematic reviews of randomised control trials with adults were included. The screening process and quality assessment with AMSTAR-2 were conducted independently by the two authors. The extracted data was synthesised narratively. In addition, four meta-analyses were pooled in a panoramic meta-analysis.

**Results:**

Of 187 reviews identified in the search, 19 were included, comprising 205 unique trials. Four meta-analyses on the effects of booster sessions, behaviour change techniques, goal setting and motivational interventions showed a significantly small overall effect (SMD 0.24, 95% CI 0.13, 0.34) and no statistical heterogeneity (*I*^2^ = 0%) in the panoramic meta-analysis. Narrative synthesis revealed substantial clinical and methodological diversity. In total, the certainty of evidence is low regarding the efficacy of the investigated interventions and techniques on adherence, due to various methodological flaws. Most of the RCTs that were included in the reviews analysed cognitive and behavioural interventions in patients with musculoskeletal diseases, indicating moderate evidence for the efficacy of some techniques, particularly, booster sessions, supervision and graded exercise. The reviews provided less evidence for the efficacy of educational and psychosocial interventions and partly inconsistent findings. Most of the available evidence refers to short to medium-term efficacy. The combination of a higher number of behaviour change techniques was more efficacious.

**Conclusions:**

The overview of reviews synthesised various potentially efficacious techniques that may be combined for a holistic and patient-centred approach and may support tailoring complex interventions to the patient’s needs and dispositions. It also identifies various research gaps and calls for a more holistic approach to define and measure adherence in physiotherapy.

**Systematic review registration:**

PROSPERO CRD42021267355.

**Supplementary Information:**

The online version contains supplementary material available at 10.1186/s13643-024-02538-9.

## Background

Adherence to physiotherapeutic1 treatment and recommendations is crucial to achieving the planned goals and desired effects [1, 2]. This is because the desired effects are usually only achieved in the long term if the recommended treatment and home-based exercises are carried out regularly. However, non-adherence in physiotherapy can be as high as 70%, particularly in unsupervised home exercise programmes [1, 3] and may differ among medical conditions [4]. The World Health Organization defines adherence to therapy as ‘the extent to which a person’s behaviour—taking medication, following a diet and/or executing lifestyle changes, corresponds with agreed recommendations from a health care provider’ [5]. Long-term adherence often requires lifestyle changes, which can be supported by behaviour change techniques (BCTs). BCTs are considered the ‘active, replicable and measurable component of any intervention designed to modify behaviour’ ([6],cf. [7]). BCTs are defined and operationalised in the behaviour change taxonomy [8], based on theoretical underpinnings and a Delphi study. Theoretical models to explain (non-)adherence and (a) motivation as well as techniques to promote behaviour change have been extensively studied in health and exercise psychology [9–11]. Rhodes and Fiala [12] argue that despite several strong psychological theories that have been developed to explain behaviour, few provide guidance for the design and development of interventions. Furthermore, theories may not be equally applicable to all behavioural domains, therapeutic regimes and settings. For example, the factors determining adherence to (passive) medication use differ from those influencing adherence to (active) physical therapies and exercise behaviour (cf. [5]). This review specifically addresses the domain of physiotherapy and therapeutic exercise.

Existing reviews of predictive studies identified factors influencing adherence positively or negatively, showing the predominately conflicting and low evidence of a wide range of predictive factors for adherence [1, 2, 13]. Moderate to strong evidence was shown for some factors, referring to previous (adherence) behaviour and treatment experiences, physical activity level, social support and psychosocial conditions, number of exercises and motivational dispositions. Such predictive studies have identified the possible targets for intervention but do not provide evidence on the efficacy of interventions. In contrast, randomised control trials (RCTs) are recognized as the preferred study design for investigating the efficacy of interventions. Thus, this overview of reviews^1^ aimed at providing a synthesis of reviews that examined RCTs, allowing for the discussion of the efficacy of different interventions and BCTs on adherence-related outcomes.

There are numerous reviews on adherence to physiotherapy and (home-based) exercise, and on BCTs to increase physical activity levels, therapeutic exercise or self-organised exercise [1–3, 14–18]. Yet, no systematic overview of reviews has been identified that specifically synthesised the efficacy of interventions and techniques to enhance adherence to physiotherapy.

### Objectives and research questions

Therefore, the aim of this overview of reviews was to synthesise the evidence on the efficacy of interventions and techniques on adherence in physiotherapy, to explore heterogeneity regarding the theoretical underpinnings, types of interventions used, and the adherence-related measures and outcomes reported, and finally to identify research gaps. Thus, the primary research question is the following: How efficacious are interventions and techniques in increasing adherence to physiotherapy? Secondary research questions are as follows: What types of intervention and behaviour change techniques were investigated? Which theoretical underpinning was reported? How was adherence defined and related outcomes measured?

## Methods

This overview of reviews is guided by the research questions and aligns with the common purposes of overviews [19, 20] and the three functions for overviews proposed by Ballard and Montgomery [21], i.e. to explore heterogeneity, to summarize the evidence and to identify gaps. This overview approach is appropriate for addressing the research questions specified above by exploring different types of interventions and behaviour change techniques and by synthesising the evidence from systematic reviews of RCTs on their efficacy. The review protocol was registered ahead of the screening process in PROSPERO (reg.nr. CRD42021267355). The only deviations from the registration were that we excluded reviews of only cohort studies, due to the already broad heterogeneity of intervention and outcome measures, and that we additionally performed a panoramic meta-analysis.

### Information sources, search strategy and eligibility criteria

The search in seven databases, PEDro, PubMed, Cochrane Library, Web of Science, Scopus, PsycInfo and CINAHL (Cumulative Index to Nursing and Allied Health Literature), was last updated on January 31, 2023. The search strategy was structured according to the PICOS (Population, Intervention, Comparison, Outcome and Study Type) scheme. The search terms related to physiotherapy and motivation or behaviour change and adherence and effectiveness/efficacy (details on the searches are listed in Additional file 1). A filter was applied limiting the search to (systematic) reviews. No publication date restrictions were applied.

Table 1 outlines the study inclusion and exclusion criteria. Only studies published in peer-reviewed journals were included. The review addressed adult patients, with any illness, disease or injury, and thus excluded studies on healthy populations. Reviews in the field of physiotherapy, physical therapy or the therapeutic use of exercise or physical activity were included if they investigated adherence as a primary outcome. Studies measuring adherence as a secondary outcome were excluded as they do analyse interventions that were not primarily designed to promote adherence and thus are outside the scope of this overview. Reviews that analysed only studies on digital apps or tools (e.g. virtual reality, gamification, exergames or tele-rehabilitation) were excluded from this overview, as they were outside of the scope of this overview. Only systematic reviews that appraised RCTs were included. Reviews appraising RCTs and other study designs were included if RCT results could be extracted separately. Systematic reviews are in our understanding literature reviews of primary studies with a comprehensive description of objectives, materials and methods; considering the risk of bias and confidence in the findings; and reporting according to the PRISMA statement [22–24]. Adherence is defined as the extent to which a person’s behaviour corresponds with treatment goals, plans or recommendations [5]. Related terms used in the literature are compliance, maintenance, attendance, participation and behaviour change or lifestyle modification and were thus included in the search strategy.

**Table 1:** Inclusion and exclusion criteria

	**Inclusion criteria**	**Exclusion criteria**
Study design	Systematic reviews of RCTs Mixed methods review that presents results from RCTs separately	Any study design other than a review Reviews of only non-RCTs
Language	Articles in any language, including an English title and abstract	No restrictions
Year of publication	Any	No restrictions
Type of publication	Peer-reviewed and indexed in the selected databases	Not peer-reviewed
Population	Adults Clinical patients/inpatients Extramural patients/outpatients	Minors (< 18 years old) People without injury, disorder or illness
Intervention (context)	Physiotherapy/physical therapy/manual therapy Therapeutic exercise/exercise therapy Home-based programme	Competition-related exercise and sport Non-therapeutic exercise Only digital tools (exergames, virtual reality, tele-reha, mobile apps)
Intervention techniques (process)	Motivation/motivational intervention, strategies and techniques Health education, counselling Behaviour change techniques Motivational interviewing Patient-centeredness/therapeutic alliance/autonomy-support Supportive climate/environment	
Outcome	Adherence/compliance/maintenance Lifestyle modification/behaviour change	

### Screening and selection process

**Fig. 1:**
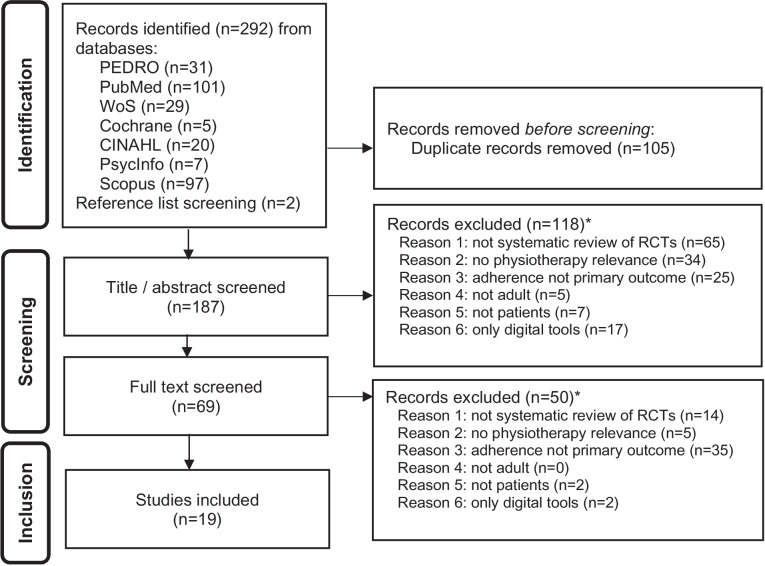
Flow diagram, based on PRISMA [24] and PRIOR [29] guidelines. Legend: *Multiple reasons for exclusion were possible

Author CL conducted the search in the seven different databases and removed duplicates, using the Zotero bibliography management tool. Following this, authors CL and PP both independently screened the titles and abstracts of the resulting sources (see Fig. 1 Flow diagram). After removing the excluded studies, PP and CL independently screened the remaining full texts in an unblinded standardised manner. Reasons for exclusion were noted in a screening spreadsheet. Any discrepancy was discussed, verified and resolved by consensus.

### Data collection process and data items

Data extraction was done by CL after agreeing with PP on the criteria. A spreadsheet was created with the following data extraction components: (i) objectives and main topic of the review; (ii) study design(s) and number of studies included and excluded; (iii) search strategies (incl. PICO); (iv) population including diagnosis, sample sizes and age; (v) intervention and comparison, theoretical foundations and models used for designing the intervention; (vi) time frames, including follow-up; (vii) adherence-related outcome and outcome measures; (viii) key findings; (ix) analysis of primary studies (meta-analytical, other statistical or narrative analysis); and (x) tools used for the quality assessment, risk of bias and evidence grading. Primary outcomes on adherence included, adherence rates or categories, engagement, attendance and participation, and accomplished physical activity levels. PP verified the data extraction results. The data was extracted as reported in the systematic reviews, then reformatted and displayed in the tables and used for the narrative synthesis.

### Assessment of risk of bias across reviews

Systematic reviews of RCTs are ranked highest in the evidence level [25], but are subjected to risk of bias (RoB). In an overview of reviews of systematic reviews, there are further risks of bias, in addition to those deriving from the primary studies and those deriving from the review of those studies. Particularly, the overlap of reviews regarding the included individual studies may bias the findings. According to the purpose of this overview, i.e. to synthesise the wide range of interventions and behaviour change techniques used to promote adherence and to summarise the evidence of their efficacy, the overlap of reviews regarding intervention or population was not an exclusion criterion. For considering the overlap of primary studies among the reviews, CL extracted the primary RCTs from the included reviews, identified the unique trials and compared the frequency of their use across the reviews (see results overlap of review and Additional file 2). Furthermore, where two or more reviews provided findings on the same technique (e.g. on the efficacy of behavioural graded activities), the overlap of primary studies was assessed specifically for that finding. If the evidence came from the same study, this was taken into account and marked accordingly in Table 5 to avoid double counting and overestimation of evidence.

### Assessment of risk of bias within the reviews

CL and PP independently assessed the quality and risk of bias of the systematic reviews included, using the AMSTAR-2 tool [26]. Any discrepancy was discussed and resolved by consensus. AMSTAR (A MeaSurement Tool to Assess systematic Reviews) was developed to evaluate systematic reviews of randomised trials. The AMSTAR-2 revision enables a more detailed assessment of systematic reviews which may also include non-randomised studies of healthcare interventions. The applied AMSTAR-2 checklist consists of 16 items, whereof seven are classified as critical, and the appraisal results in an overall confidence rating distinguishing between critically low, low, moderate or high [26]. In addition, the overall confidence in the review was stipulated by the number of positive assessments in relation to the applicable domains (depending if meta-analysis was performed or not) and considering whether an item represents a critical domain or not [26].

### Synthesis methods

#### Panoramic meta-analysis

Among the included reviews, there were four meta-analyses [7, 16, 27, 28], which were pooled as a panoramic meta-analysis based on the reported effect sizes and standard errors using IBM SPSS Version 29 (IBM Corp., Armonk, NY, USA). All four meta-analyses used the standardized mean difference as effect size. Standard errors were calculated from the reported 95% CI as $\frac{\mathrm{upper bound }-\mathrm{ lower bound}}{3.92}$. Inverse variance was used to weight the meta-analyses, statistical heterogeneity was assessed by *I*-squared and a fixed-effects model was selected based on the absence of statistical heterogeneity of true effects. Eisele et al. [7] included 15 primary trials that examined the effect of BCTs on physical activity adherence. They pooled results for medium-term (3–6 months) and long-term (7–12 months) interventions, from which we selected the medium-term model that best matched the eligibility criteria of the other included meta-analyses. Levack et al. [27] included nine primary trials that examined the effect of goal-setting strategies on engagement in rehabilitation. Among models with other outcomes, we selected this model because it best matched the aim of this overview, and it was most consistent with the outcomes of the other included meta-analyses. McGrane et al. [28] included six primary trials, representing 378 subjects that examined the effects of motivational interventions on physiotherapy session attendance. They reported another model with perceived self-efficacy as an outcome, but we selected the attendance model because it best matched the aim of this overview, and it was most consistent with the outcomes of the other included meta-analyses. Nicolson et al. [16] included two primary trials that examined the effect of booster sessions on self-rated adherence. Results were summarized by a forest plot and publication bias was assessed graphically by a funnel plot, although the small number of individual meta-analyses included limits its interpretability. Alpha was set at 0.05.

#### Narrative synthesis

The narrative synthesis was performed by CL in constant dialogue with and verification of PP. Guided by the research questions, the narrative synthesis of the extracted data was manifold. First, we explored the heterogeneity of interventions, measures and adherence-related outcomes across and within the reviews using the data extraction table. Definitions and measures of adherence were compared among the reviews and discussed. Second, analysis of the descriptions of the interventions and their respective components/techniques, their theoretical underpinning and their objectives was used to classify the interventions according to different types of intervention, namely the informational/educational, the cognitive/behavioural/motivational and the relational/psychosocial intervention. Consequently, for each type of intervention, the results on the efficacy were narratively synthesised. In addition, reported differences in efficacy among medical conditions, theoretical underpinnings and physiotherapeutic settings were summarised based on the data extraction table. Third, the results on the efficacy of the interventions and BCTs were further summarised in a table and then restructured according to the evidence level as reported in the systematic reviews and the confidence in the reviews as analysed by the AMSTAR-2. Therefore, the levels of evidence were extracted as reported in the reviews, which are based on different evidence appraisal schemes: GRADE (high, moderate, low, very low certainty of evidence), Cochrane Collaboration Back Review Group Evidence Levels (strong, moderate, conflicting, limited, no evidence) and self-developed tools. Afterwards, they were compared for the respective intervention/technique across the relevant reviews, considering the confidence in the review and the comprehensiveness of the review as well. The levels of evidence are presented in the table with the categories high, moderate, low and very low. The efficacy supported by the evidence is also based on the results reported in the reviews. In case of overlapping reviews or discrepancies between the reviews, the primary studies were consulted. The category *yes* refers to results of merely positive effects, and *inconsistent* refers to findings of positive and no effects of the intervention (techniques) analysed. The category *no* indicates that the intervention was not efficacious. No negative effects (i.e. favouring the control condition) were reported for the intervention (techniques) shown.

The reporting of findings followed the PRIOR reporting guideline for overviews of reviews of healthcare interventions [29].

## Results

### Study selection results

Of the 187 records screened, 19 were included (see Fig. 1). Main reasons for exclusion were *not a systematic review of RCTs* (*n* = 79), *adherence not the primary outcome* (*n* = 60), and *lack of* *physiotherapy relevance* (*n* = 39) (see Fig. 1).

### Characteristics and diversity of included reviews

The selection strategy resulted in a broad heterogeneity of included reviews. The 19 included reviews differed in their eligibility criteria of the primary studies as well, resulting in substantial clinical diversity, i.e. the inclusion of heterogenous conditions, intervention types and settings (see Table 2) and methodological diversity, i.e. the variability in study design, outcome measurements and risk of bias (see Tables 3, 4 and 5). Musculoskeletal diseases [6, 7, 17, 30–32] and pain [13, 16, 33–35] were the most investigated medical conditions. Those reviews that did not limit their search to a specific disease [12, 27, 28, 36–40] yielded predominantly studies on musculoskeletal diseases. All reviews included adults only (18 and older). One focused on elderly (65 and older) people [40] and one on older (45 and older) adults [16]. Fourteen of the 19 reviews analysed RCTs only [6, 7, 16, 17, 27, 28, 30–36, 39, 40]; one also included besides RCT cohort studies [13] and three [12, 37, 38] also included any other quantitative study design (see Table 3). Four reviews performed a meta-analysis [7, 16, 27, 28], and two studies were Cochrane Reviews [27, 35]. Four reviews [6, 7, 17, 40] analysed the use of BCTs and rated the interventions according to a BCT taxonomy [8].

**Table 2:** Study characteristics

	**Objectives**	**Population**	**Intervention**	**Outcome measurements**
[36]	To evaluate the efficacy of video-assisted patient education to modify behaviour	Adult patients with any health-related problem	IG: video-assisted patient education (audio-visual educational component as a separate intervention) CG: standard care or elaborated control condition	Behaviour change, by direct measurement, objective rating or laboratory data. Excluded self-report and questionnaire
[13]	To gain insights into the factors associated with adherence to exercise or physical activity programs to prevent or treat neck pain and low back pain	Adults with a diagnosis of neck or lower back pain	IG: exercise therapy, goal-setting intervention, motivation programme, phone surveillance, augmented feedback, communication skills training CG: low exercise, standard care	Exercise adherence (diary, self-reported questionnaire and direct observation)
[37]	To list the factors that influence adherence to home-based exercise and to recommend how to improve adherence	Any patients, no age or illness restriction	IG: home exercise program CG: any	Adherence by an exercise log and exercise behavior (frequency, walking distance and duration)
[33]	To identify factors associated with adherence to health care practitioner-prescribed home exercise	Adults (18–65) with chronic lower back pain	IG: prescribed home exercise CG: any	Adherence (any measure, incl. any scale, questionnaire, assessments of physical limitations, pain or participation)
[32]	To determine different methods used to enhance physiotherapy exercise adherence for a period of more than 12 months among patients with osteoarthritis and to report the most effective methods	Adults with lower limb osteoarthritis	IG: Intervention(s) aimed to improve adherence, compliance or engagement with exercise CG: no adherence or engagement intervention	Adherence to self-rated or self-reported, physical activity maintenance, attendance marked by trainers
[7]	To identify BCTs applied in interventions to enhance PA adherence and to investigate the efficacy of these interventions in increasing PA adherence	Adults suffering from chronic musculoskeletal conditions for at least 3 months	IG: BCTs CG: Usual care, minimal intervention, placebo or no intervention were considered eligible control conditions	Any measure of PA level or adherence to any kind of PA
[38]	To investigate whether the working alliance is related to outcome in physical rehabilitation	Any patient with/in physical rehabilitation (included back pain)	IG: Physical rehabilitation for 4 to 16 weeks, administered by a physical therapist. CG: any comparable group	Adherence, attendance, activities of daily living, working alliance inventory
[34]	To systematically review the evidence for health coaching and describe the diversity of health coach training and interventions	Adults aged 18 or over with lower back pain with or without leg pain (sciatica)	IG: any health coaching intervention, but excluded cognitive behavioural therapy (CBT). CG: No limitations were placed on comparison interventions	Exercise compliance, physical activity, self-efficacy for regular physical activity
[35]	To assess the effects of interventions to improve adherence to exercise and physical activity for people with chronic musculoskeletal pain	People with chronic musculoskeletal pain, including adults with pain for 3 months and over in the axial skeleton or large peripheral joints	IG: any interventions delivered in primary, outpatient or community care that aimed to improve adherence to exercise or physical activity. CG: Other interventions with the same aim, control groups that receive no intervention or other exercise interventions	Adherence to advised or prescribed exercise or physical activity; and changes in general exercise or physical activity behaviour
[39]	To determine the evidence regarding the efficacy, use and modes of action of goal planning in clinical rehabilitation	Adult patients with any post-acute or chronic disabling conditions (excluding congenital or developmental origin and those relating to psycho-active substance use, or mental illness)	IG: Intervention that involves an aspect of goal planning or approach to goal planning. CG: adequate control of variables associated with the approach to goal planning (without introducing other additional variables to the ‘control’ group or to the ‘goal planning’ group)	Adherence to treatment regimes over the duration of a whole rehabilitation programme
[27]	To assess the effects of goal setting and strategies to enhance the pursuit of goals on improving health outcomes	People receiving rehabilitation for disability acquired in adulthood (included studies on musculoskeletal disorders, brain injury, chronic pain, mental health conditions, and cardiovascular disease)	IG: Studies that investigated the effects of establishing and negotiating rehabilitation goals, with or without strategies to enhance goal pursuit CG: no goal setting;’usual care’; another structured approach	Activity outcomes (e.g. activities of daily living, mobility); engagement in rehabilitation (e.g. adherence, patient motivation, self-efficacy); Individual goal attainment Any measurement
[28]	To evaluate the evidence for the efficacy of adding motivational interventions to traditional PT to increase physical activity and short- and long-term adherence to exercise prescriptions	Any adult	IG: Motivational interventions (incl. psychological strategies, theory-based instructional manuals, Internet-based behavioural programmes and relapse prevention and reinforcement strategies). CG: Standard care, PT only, exercise only	All measures of adherence were included
[30]	To identify strategies to improve adherence to musculoskeletal outpatient treatment	Adults with mechanical musculoskeletal dysfunctions of an acute, chronic or postoperative nature	IG: interventions to improve treatment adherence; administered mainly by physical or exercise therapists. (1) use of supporting materials and (2) cognitive-behavioural interventions. CG: Standard care, exercise only	Attendance, adherence, regularity of exercising at home, frequency of physical activity, exercise behaviour, training frequency and duration
[16]	To evaluate whether interventions aimed at increasing adherence to therapeutic exercise increase adherence greater than a contextually equivalent control	Adults 45 years or older with chronic (> 3 months) lower back pain and/or hip/knee osteoarthritis	IG: Any intervention that aimed to improve adherence to therapeutic exercise. CG: Therapeutic exercise comparable to the intervention arm, without specific adherence strategy or intervention	Any quantitative measure of exercise adherence, including numerical rating scales and logbook/diary measures
[31]	To systematically review educational or psychoeducational interventions for patients with rheumatoid arthritis focusing on long-term effects	Patients with rheumatoid arthritis	IG: Patient education interventions aiming to improve knowledge, health behaviour or skills or to influence the psychological or physical health status CG: No intervention, PT, waiting list	Compliance, performance, physical activity, self-efficacy at pre-, post- and follow-up
[12]	To review the available evidence for (1) the modifiable factors associated with adherence to physical therapy recommended exercise and (2) the efficacy of intervention efforts	Any adult	IG: Individuals receiving physical therapy treatment with exercise/physical activity prescribed or recommended by a physical therapist (at home or in a supervised environment) CG: any	Measures of behavioural adherence
[40]	To assess the efficacy of interventions used to improve exercise adherence in older people, and to evaluate the behavioural change techniques underpinning them	Population of 65 years or older AND community-dwelling (excluding those with a diagnosis of dementia or cognitive impairment)	IG: intervention(s) aiming to improve adherence, compliance, concordance to or engagement with exercise. CG: either no adherence, compliance, concordance or engagement intervention; another adherence, compliance, concordance or engagement intervention	Adherence, compliance, concordance to or engagement with exercise
[6]	To investigate whether BCTs can influence adherence to home exercise	Upper extremity musculoskeletal disorders	IG: any home exercise programme, alongside a BCT designed to increase exercise adherence CG: any	Exercise adherence self‐reported by patients
[17]	To identify and evaluate the efficacy of behavioural change techniques (BCTs) within physiotherapy interventions to increase physical activity (PA) adherence in patients with lower limb OA	Adult participants with lower limb osteoarthritis (hip and/or knee), diagnosis through self-report of symptoms or imaging	IG: The physiotherapist delivered intervention including at least one BCT specifically enhancing PA adherence away from the clinic e.g. activity diary/pedometer or follow-up care that made it unique from its comparator group. CG: Other ‘active’ or ‘placebo’ interventions, ‘no treatment’ or ‘usual care’	Primary outcomes: physical activity or adherence measures Secondary outcomes: pain, function, quality of life, self-efficacy, any adverse events

Legend: *IG*, intervention/experimental group; *CG*, control/comparator group; *BCT*, behaviour change techniques; *PA*, physical activity; *PT*, physiotherapy

**Table 3:** Critical appraisal of the included systematic reviews with the AMSTAR-2 tool

**Item**	[36]	[13]	[37]	[33]	[32]	[7]	[38]	[34]	[35]	[39]	[27]	[28]	[30]	[16]	[31]	[12]	[40]	[6]	[17]
1. Did the research questions and inclusion criteria for the review include the components of PICO?	1	1	0	0	1	1	0	1	1	1	1	1	0	1	0	0	1	1	1
2.^**a**^Did the report of the review contain an explicit statement that the review methods were established prior to the conduct of the review and did the report justify any significant deviations from the protocol?	**0**	**0**	**0**	**0**	**½**	**1**	**0**	**0**	**1**	**0**	**1**	**0**	**0**	**0**	**0**	**0**	**½**	**½**	**1**
3. Did the review authors explain their selection of the study designs for inclusion in the review?	0	0	0	0	0	0	0	0	1	0	0	0	0	0	0	0	0	0	0
4.^**a**^Did the review authors use a comprehensive literature search strategy?	**½**	**½**	**½**	**½**	**½**	**½**	**1**	**½**	**1**	**½**	**1**	**0**	**½**	**½**	**0**	**½**	**½**	**½**	**1**
5. Did the review authors perform study selection in duplicate?	1	1	0	1	1	1	0	1	1	1	1	1	1	1	0	0	1	1	1
6. Did the review authors perform data extraction in duplicate?	0	1	0	1	1	0	1	0	1	1	1	1	1	1	1	0	1	1	1
7.^a^Did the review authors provide a list of excluded studies and justify the exclusions?	**0**	**0**	**0**	**0**	**0**	**0**	**0**	**0**	**1**	**0**	**1**	**0**	**0**	**0**	**0**	**0**	**0**	**0**	**0**
8. Did the review authors describe the included studies in adequate detail?	0	**½**	**½**	0	0	1	0	1	**½**	**½**	1	**½**	**½**	1	**½**	**½**	1	1	1
9.^**a**^Did the review authors use a satisfactory technique for assessing the RoB in individual studies that were included in the review?	**0**	**1**	**0**	**1**	**1**	**1**	**0**	**1**	**1**	**1**	**1**	**1**	**1**	**1**	**1**	**0**	**1**	**1**	**1**
10. Did the review authors report on the sources of funding for the studies included in the review?	0	0	0	0	0	0	0	0	0	0	1	0	0	0	0	0	0	0	0
11.^**a**^If meta-analysis was performed did the review authors use appropriate methods for statistical combination of results?	**NA**	**NA**	**NA**	**NA**	**NA**	**1**	**NA**	**NA**	**NA**	**NA**	**1**	**1**	**NA**	**1**	**NA**	**NA**	**NA**	**NA**	**NA**
12. If meta-analysis was performed, did the review authors assess the potential impact of RoB in individual studies on the results of the meta-analysis or other evidence synthesis?	NA	NA	NA	NA	NA	1	NA	NA	NA	NA	1	0	NA	0	NA	NA	NA	NA	NA
13.^**a**^Did the review authors account for RoB in individual studies when interpreting/discussing the results of the review?	**0**	**1**	**0**	**0**	**0**	**1**	**0**	**1**	**1**	**0**	**1**	**1**	**0**	**1**	**0**	**0**	**1**	**1**	**0**
14. Did the review authors provide a satisfactory explanation for, and discussion of, any heterogeneity observed in the results of the review?	1	1	0	0	0	1	0	1	0	0	1	0	0	0	0	0	0	0	1
15.^**a**^If they performed quantitative synthesis did the review authors carry out an adequate investigation of publication bias (small study bias) and discuss its likely impact on the results of the review?	**NA**	**NA**	**NA**	**NA**	**NA**	**1**	**NA**	**NA**	**NA**	**NA**	**1**	**0**	**NA**	**1**	**NA**	**NA**	**NA**	**NA**	**NA**
16. Did the review authors report any potential sources of conflict of interest, including any funding they received for conducting the review?	1	1	1	1	1	1	0	0	1	1	1	1	0	1	0	0	1	1	1
Overall confidence rating	CL	L	CL	CL	CL	M/H	CL	L	H	CL	H	L	CL	M	CL	CL	L	L	L

Legend: ^a^Critical domains; *1*, yes; *2*, no; *NA*, not applicable; *CL*, critically low; *L*, low; *M*, moderate; *H*, high confidence in the review

**Table 4:** Main results of the reviews

	**Study design** (number of included studies)	**Main results**
[36]	SR of RCTs (20)	• No clear evidence for the efficacy of video-assisted patient education in modifying behaviour. Ten of the 20 included articles reported a difference between experimental/treatment conditions versus control conditions in the expected direction. No difference in the overall score (5.8 ± 1.1 versus 5.1 ± 1.9; Mann–Whitney-*U* test: *p* = 0.631) between the studies that did report a behavioural change compared with studies that did not report a change • Didactic information may increase health literacy but is not sufficient to modify health-related behaviour • Videos that only provide spoken or graphically presented health information are inappropriate tools to modify patient behaviour. Videos with a narrative format seem to be a powerful education tool
[13]	SR of RCTs with follow-up (9) or cohort study (8)	• Nine RCTs showed moderate-quality evidence for the association between exercise adherence and self-efficacy • Low-quality evidence with serious inconsistency and imprecision for the association between exercise adherence and counselling, goal setting, phone surveillance and communication skills training
[37]	SR of quantitative studies: SR (4); RCTs (2); cross-sectional (1); prospective study (7)	• One RCT found no differences among intervention groups • One RCT revealed a significant treatment effect, with subjects in the intervention group (5-month exercise + motivational intervention programme) exercising more frequently (effect size: 0.54), for longer duration (effect size: 0.50) and walking greater distance (effect size: 0.52)
[33]	SR of RCTs (11 studies on 9 trials) and non-RCTs (3)	• Two high-quality, six medium-quality and one low-quality RCT • No factor was found to be strongly associated with adherence • Moderate evidence that supervision (2 studies; *n* = 193) and participation in an exercise program (4 studies; *n* = 613), and the use of a general behaviour change program, incorporating motivational strategies (3 studies; *n* = 267) were associated with adherence to home exercise • Limited evidence that participation in the development of an exercise program (1 study; *n* = 48), participating in a behavioural program to enhance adherence (1 study; *n* = 48), use of positive reinforcement (1 study *n* = 40), participation in a Pilates-style program (1 study *n* = 53) and regular therapist follow-up (1 study; *n* = 48) were associated with adherence to home exercise
[32]	SR of RCTs (5)	• While one RCT did show differences between the intervention and comparator groups on long-term adherence, two RCTS did not yield differences and two RCTs showed only tendencies that were not statistically significant or only significant in the short term • Behavioural graded exercise with booster sessions improved adherence
[7]	SR of RCTs (22) Meta-analysis	• A small medium-term overall effect (pooled SMD 0.20, 95% CI 0.08–0.33, *p* < 0.01) and no long-term effect of interventions comprising BCTs in enhancing PA adherence (pooled SMD 0.13, 95% CI 0.02–0.28, *p* = 0.09) • Interventions using a greater number of BCTs (between-group difference 8 BCTs) attained a higher effect (pooled SMD = 0.29, 95% CI 0.19–0.40, *p* < 0.001) than interventions applying a lower number of BCTs (between-group difference < 8 BCTs; no effect; pooled SMD = 0.08, 95% CI − 0.11–0.27, *p* = 0.41) • Sensitivity analyses considering only studies with PEDro scores ≥ 6 revealed slightly lower effect sizes, but no change in statistical significance (medium-term effect: pooled SMD = 0.16, 95% CI 0.04–0.28, *p* < 0.01; long-term effect: the same studies included, no change; BCTs high: pooled SMD = 0.26, 95% CI 0.16–0.37, *p* < 0.001; BCTs low: pooled SMD = 0.09, 95% CI 0.12 to 0.30,* p* = 0.39). Low risk of publication bias. Heterogeneity of study outcomes was low to moderate (*I*^2^ between 0 and 49%)
[38]	SR of RCTs (3) cohort study (9) or cross-sectional (1)	• The results indicate a consistent positive correlation between the therapist-patient alliance and treatment outcomes of pain, disability, physical and mental health and satisfaction with treatment
[34]	SR of RCTs (4)	• The body of evidence was graded as very low. One RCT found significant improvements in exercise compliance in favour of the health coaching group at both follow-up points with a large and moderate SMD (1.3 and 1.26) • All included studies are based on health-coaching interventions on the transtheoretical model of change; however, the content of counselling programmes varied between studies and measures of treatment fidelity were inconclusive
[35]	SR of RCTs (42)	• Of the 18 trials that showed improved adherence to exercise, only eight also showed significant improvements in at least one clinical outcome. One trial showed a significant difference in exercise adherence between two different types of exercise training programmes, but no difference in clinical outcomes. In another trial that compared different types of exercise, significant differences in adherence measures did not correspond with a significant difference in clinical outcomes. In total GRADE: Moderate, inconsistent evidence (− 1); Silver • Exercise type does not appear to be an important factor in order to improve exercise adherence. (GRADE: Moderate (inconsistent interventions (− 1)); Silver). Evidence for water-based exercise is conflicting (GRADE: Low (moderate quality (− 1) and inconsistent results (− 1)); Silver). Supervised exercise is more effective for improving weekly training frequency than unsupervised exercise. (GRADE: Moderate (moderate quality (− 1)); Silver). Individual exercise is more effective than group exercise for improving attendance at exercise classes. (GRADE: Moderate (moderate quality (− 1)); Silver). Supplementing a home exercise programme with group exercise may increase overall physical activity levels. (GRADE: Moderate (moderate quality (− 1)); Silver) • Therapeutic programmes that specifically address exercise adherence are effective in improving the frequency/duration of exercise, and attendance at sessions. (GRADE: Moderate (moderate quality (− 1)); Silver). The addition of transtheoretical model-based counselling to physiotherapy is not more effective than physiotherapy and a sham intervention (GRADE: High (high quality); Silver). Self-management programmes improve exercise frequency compared to waiting lists or no-intervention control groups. (GRADE: Moderate (moderate quality (− 1)); Silver). Graded activity is effective in improving adherence to a home exercise programme. (GRADE: Moderate (moderate quality (− 1)); Silver) • The addition of interventions based on CBT to physiotherapy programmes may be effective for people with whiplash-associated disorders. (GRADE: Moderate (moderate quality (− 1)); Silver); however, evidence suggests that adding CBT-based approaches to physiotherapy programmes is not effective in improving exercise adherence for other chronic musculoskeletal conditions. (GRADE: Moderate (moderate quality (− 1)); Silver) • There is conflicting evidence on whether interventions that significantly improve adherence also significantly improve clinical outcome measures in comparison to a control/comparison group (GRADE: Moderate (inconsistent evidence (− 1)); Silver)
[39]	SR of RCTs (19)	• Some limited evidence exists that goal planning can improve patients’ adherence to treatment regimes • Strong evidence that specific, difficult goals can improve immediate patient performance in some clinical contexts • Evidence regarding any generalizable effect of goal planning on improving outcomes following rehabilitation programmes is inconsistent at present
[27]	SR of RCTs (39): 27 RCTs, 6 cluster-RCTs, and 6 quasi-RCT Meta-analysis	• Very low-quality evidence that including any type of goal setting in the practice of adult rehabilitation is better than no goal setting for health-related quality of life or self-reported emotional status (8 studies; *n* = 446; SMD 0.53, 95% CI 0.17 to 0.88) and self-efficacy (3 studies; *n* = 108; SMD 1.07, 95% CI 0.64 to 1.49) • Very low-quality evidence that more structured goal setting results in higher patient self-efficacy (2 studies; *n* = 134; SMD 0.37, 95% CI 0.02 to 0.71) and low-quality evidence for greater satisfaction with service delivery (5 studies; *n* = 309; SMD 0.33, 95% CI 0.10 to 0.56) • Low evidence that goal planning/setting improves engagement in rehabilitation (motivation, involvement and adherence) over the duration of the programme (9 studies; 369 participants; SMD 0.30, 95% CI -0.07 to 0.66) • Three studies in this group reported on adverse events (death, re-hospitalisation or worsening symptoms), but insufficient data are available to determine whether structured goal setting is associated with more or fewer adverse events than usual care • The evidence is inconclusive regarding whether goal setting results in improvements in social participation or activity levels, or levels of patient engagement in the rehabilitation process. The best of this evidence appears to favour positive effects for psychosocial outcomes (i.e. health-related quality of life, emotional status and self-efficacy) rather than physical ones
[28]	SR of RCTs (14) and meta-analysis	• No significant difference (*p* = 0.07) in *exercise attendance* between the groups (SMD 0.33, 95%CI -0.03 to 0.68, *I*^2^ 62%) • Perceived *self-efficacy* results were pooled from six studies (*n* = 722), and a significant difference was found between the groups in favour of the interventions (fixed effects model, SMD 0.71, 95% CI 0.55 to 0.87, *I*^2^ 41%) • The results for *levels of activity limitation* were pooled (*n* = 550), and a significant difference was found between the groups in favour of the interventions (REM, standardised mean difference − 0.37, 95% confidence interval − 0.65 to − 0.08, *I*^2^ 61%) • Due to heterogeneity in the different approaches applied, it was not possible to complete a subgroup analysis to determine the most effective method • The narrative review indicates that CBT, social cognitive theory, motivational interviewing and self-determination theory have a positive effect on physical activity, self-efficacy, activity limitation, attendance and other proxy measures for adherence
[30]	SR of RCTs (6 – relating to 5 trials)	• Moderate evidence from one high-quality study (*n* = 93) that a motivational cognitive-behavioural (CB) programme can improve attendance at exercise-based clinic sessions • Conflicting evidence from two low-quality studies (*n* = 136) that supporting material increases short-term adherence (< 6 months) with exercise. One study provides limited evidence of no effectiveness for individualised exercise videos • Strong evidence that adherence strategies are not effective at improving long-term adherence with home exercise • There is conflicting evidence from three high-quality studies (*n* = 310) that CB interventions are effective at increasing short-term adherence to exercise • There is strong evidence from three high-quality studies (*n* = 310) that CB interventions are not effective at enhancing long-term (6 months) adherence with exercise
[16]	SR of RCTs (9) and meta-analysis	• Meta-analysis with two studies provides moderate-quality evidence that booster sessions with a physiotherapist assisted people with hip/knee osteoarthritis to better adhere to therapeutic exercise (SMD 0.39, 95% CI 0.05 to 0.72, *z* = 2.26, *p* = 0.02, *I*^2^ 35%) • Four studies, evaluating strategies that aimed to increase motivation or using behavioural graded exercise, reported significantly better exercise adherence (SMD = 0.26–1.23) • In contrast, behavioural counselling, action coping plans and/or audio/video exercise cues did not improve adherence significantly • Two studies involving motivation programmes targeting increasing self-efficacy through positive reinforcement and education reported statistically significant differences between intervention and control group adherence and effect sizes ranging from large (SMD = 1.23) at short-term follow-up to small to medium (SMD = 0.44) at long-term follow-up • Behavioural counselling, focusing on readiness to change did not improve adherence
[31]	SR of RCTs (11)	• The 7 educational programs mainly improved knowledge and compliance in the short and long term, but there was no improvement in health status • All 4 psychoeducational programs improved coping behaviour in the short term, 2 of them showing a positive long-term effect on physical or psychological health variables • Methodologically better-designed studies had more difficulties demonstrating positive outcome results
[12]	SR of RCTs (7), cohort studies (5) and cross-sectional (1)	• Three RCTs on CB studies showed null findings and negligible effect sizes; one RCT showed no difference across experimental and control groups during the first 11 weeks, but significant and small-medium effect size differences in favour of the cognitive-behavioural intervention 5 and 11 months later. The latter used a heavier emphasis on coping (i.e. overcoming anticipated barriers) and action planning (i.e. how, when and where) • Conflicting results concerning the intervention medium (clinical or home-based; verbal, audio/video-cased or written) • Adherence in the trials was uniformly high, suggesting very motivated study participants, and thus questions the generalizability and the probability to find significant differences for the various mediums and interventions
[40]	SR of RCTs (11)	• One study with a high risk of bias showed no significant difference in mean exercise adherence, comparing exercise instruction given in audio and video format in addition to written instructions • Feedback and monitoring: One study with a high risk of bias showed a significant difference between the number of exercise sessions completed between a group that received individual graphic feedback related to their exercise goal and a control group at 24 weeks (*p* < 0.01). One study with a high risk of bias found that the telecommunication and community-based groups had significantly higher results for time exercising and attendance rate compared with home exercise (*p* < 0.01). One study with a high risk of bias showed more compliance with recommendations in the group with weekly exercise and motivation classes lasting 6 months compared to the written and verbal exercise advice group (*p* < 0.012) • Social support: One study, with a high risk of bias, found a short-term difference in minutes of exercise undertaken, between the intervention (weekly phone calls and one home visit over a 3-month period) and control at 20 weeks (*p* < 0.05) but not at 1-year follow-up. Three other studies showed no differences regarding social support through peer support or supervision • Goals and planning: One study, with a high risk of bias, found no significant differences in adherence rates at 3 and 6 months between a structured educational counselling booster session (given over the phone, or face to face, and in relation to the individualized goals) compared to usual care • Four studies used social learning theory, socioemotional selectivity theory, cognitive behavioural theory or self-efficacy theory
[6]	SR of RCTs (6)	• Three of the six included studies found significant differences in adherence levels after the use of BCTs. Two of these studies had a high risk of bias • There is some evidence to show that social support may be relevant in influencing adherence and that behavioural feedback, goals and planning do not. However, given that each intervention group received multiple BCTs, it is difficult to state the effect of any single technique on adherence levels
[17]	SR of RCTs (24)	• Overall, the BCTs with efficacy ratios of 100% across all measured time frames were 1.8 *behavioural contract*, 2.7 *Feedback on outcomes of behaviour* and 10.3 *non-specific reward* • Eight BCTs had a short-term efficacy ratio of 50% in at least 2 outcome domains. Of these, 3 BCTs were from the *goals and planning* and 2 from the *feedback and monitoring* hierarchies respectively • Similarly, 8 BCTs had long-term efficacy ratios of 50% with 4 BCTs coming from the *goals and planning* hierarchy • Medium-term efficacy ratios were generally lower than short- or long-term measures • Adherence outcomes had higher proportions of efficacy ratios of 50% than PA self-report or direct measures respectively. There was minimal long-term PA direct measure data available for analysis • Most BCTs had efficacy ratios of < 50% across timeframes and outcome domains, meaning that they were components of interventions that were statistically no more effective than comparator groups at optimizing PA adherence

Legend: *REM* random effects model; *SMD* standardised mean difference, *CI* confidence interval, *CB* cognitive behavioural, *BCT* behaviour change techniques

### Results of the individual reviews

The 19 reviews contained a total of 205 unique RCTs. Table 3 shows the main results of each review.

### Results of quality assessment and confidence in the reviews

The critical appraisal with the AMSTAR-2 tool (see Table 4) showed that four reviews were rated with moderate to high quality [7, 16, 27, 35], whereas all others resulted in a critically low to low overall confidence in the review. Frequent shortcomings were not explaining the reasons for the inclusion of primary study designs, and an insufficient discussion of the heterogeneity observed. Furthermore, as many reviews did not explicitly mention a pre-established, published or registered protocol or study plan, it is uncertain whether the research followed a pre-specified protocol and whether there were changes and/or deviations from it, and, if so, whether decisions during the review process may have biased the results [26].

### Risk of bias and evidence assessment within reviews

The reviews used various approaches to appraise the evidence, particularly the GRADE (Grades of Recommendation, Assessment, Development and Evaluation) system [13, 16, 26, 27], the evidence levels by the Oxford Centre for Evidence-Based Medicine [28] or the system by Cochrane Collaboration Back Review Group [published by 25,30] [31–34]. Three reviews modified existing or developed their own tool or checklist [12, 35, 36]. For the assessment of the risk of bias and/or quality of the individual studies, the reviews used the following tools: PEDro Scale [7, 13, 26, 32, 37], Cochrane Collaboration Back Review Group Quality Assessment Tool [31, 34], Cochrane Risk of Bias criteria [6, 16, 17, 27, 33, 37–39], the Delphi List [40] or modified or developed own tools [12, 35, 36].

A recurring concern regarding potential performance bias was the lack of therapist blinding, which is almost impossible to implement in this research field [7]. Attrition bias, due to low sample size or drop-outs, and measurement bias, due to the mere use of subjective measures, were also highlighted in the reviews. Another concern was the availability and selection of adequate control groups. Control groups, such as usual practice, unspecific exercise group or alternative intervention commonly include varying numbers of BCTs which must be considered when assessing and comparing contents of interventions [7]. The comparability of the intervention and control group regarding adherence-related outcomes is further hindered by poor descriptions of the intervention, uncertainty about treatment fidelity and implementation processes, varying competences and proficiency of the therapist, and the diverse translation of theoretical models and use of intervention techniques [7, 34, 39]. Rhodes and Fiala [12] pointed out that procedures of RCTs, such as several pre-screenings and measurement batteries, may lead to a potential self-selecting of only the most motivated individuals. This may limit the ability to compare intervention to the control group, as both groups are (already) highly motivated, and to detect changes, due to the already high motivation and disposition to adhere. This may explain in part, that the reviews reported many studies that failed to provide evidence for intervention efficacy on adherence. In addition, the restricted timeline (limited duration for observation and follow-up) of the studies may confound/skew the results, as drop-out may occur shortly after the end of the study and long-term adherence is not measured [12].

### Overlap of reviews

The 19 reviews included from 3 to 42 individual RCTs. In sum, the reviews included 261 RCTs (multiple publications on the same trial were counted as one; thus, the number of trials was counted), whereby 34 trials were included in various reviews (see Additional file 2, Overlap of reviews), resulting in 205 unique RCTs. Of these 34 trials included in multiple reviews, 25 were included in two different reviews. The following trials were included more than twice: Basler et al. 2007 (8x), Friedrich et al. 1998 (7x), Schoo et al. 2005 (4x), Vong et al. 2011 (4x), Asenlof et al. 2005 (3x), Bassett and Petrie 1999 (3x), Brosseau et al. 2012 (3x), Bennell et al. 2017 (3x), Gohner and Schlicht 2006 (3x) and Duncan and Pozehl 2002, 2003 (3x).

In total, the overlap of primary trials in the reviews is considered low; except among reviews [27, 39] and among reviews [12, 16, 28, 30]. Two reviews [27] and [39] were conducted by the same authors, within the same field, i.e. goal planning and setting, however with a different approach and research question. Reviews [12, 16, 28, 30] have a considerable amount of overlap. Still, each of these reviews included unique RCTs, not analysed in any of the other reviews, and they do focus on different research questions, foci and analyses. Therefore, we did not exclude an entire review due to an overlap of studies.

### Synthesis of results

The synthesis focused on answering the research questions. We began by presenting the narrative synthesis findings on how adherence was measured, what types of intervention and BCTs were investigated, and which theoretical underpinnings were reported. Afterwards, we synthesised the evidence on the efficacy of the interventions and BCTs, both meta-analytically and narratively.

#### Measures of adherence and related outcomes

The reviews included studies with a heterogeneous use, breadth and measures of adherence. Mostly, they refer to adherence as the extent to which a person’s behaviour corresponds with treatment goals, plans or recommendations ([30],cf. [5]). McLean and colleagues [30] expressed that within physiotherapy, the concept of adherence is multi-dimensional and could refer to attending appointments, following advice or undertaking prescribed exercises. The terms *adherence* and *compliance* were sometimes used interchangeably, referring to the degree of treatment attendance or accomplishment of physical activity levels, participation and recommendations, irrespective of how the treatment goals and plans were established. Yet, for definition purposes, the distinction between agreed and prescribed goals and plans was occasionally used in the reviews to distinguish *adherence* from *compliance*.

For analytical purposes, adherence was frequently dichotomised, establishing a cutoff point or percentage used to distinguish adherence from non-adherence. One was considered adherent, for example, if he/she achieved more than 70% or 80% of the targeted, recommended or prescribed sessions. Few studies graded the degree of adherence according to multi-categorical cut-off points (e.g. very low, low, moderate and high adherence). Only in one review [13], one study was named that distinguished a certain fluctuation in the adherence pattern, i.e. Dalager et al. [41] included besides the minutes exercised in a week the regularity of participation, distinguishing regular from irregular participation. Self-reported diaries, exercise logs and attendance lists were the most commonly used data recording instruments [33, 35, 37]. Adherence to home-based programmes was mainly measured with self-reported diaries, which are problematic as the only source, due to poor completion rates, and the possibility of inaccurate recall and self-presentation bias [18, 33]. Digital devices (e.g. accelerometers or pedometers) may be used additionally to measure adherence; however, their use may also be problematic, as they require certain adherence to a systematic use of the device and the mere use of the device also may increase adherence [18, 33]. One study reported the use of the Sport Injury Rehabilitation Adherence Scale (SIRAS) [42], which measures the patients’ degree and manner of participation in a session and compliance with the therapist’s instructions and plan. Thus, it does not measure adherence over a certain period of time nor adherence to recommendations or home-based exercise, but it can be used to assess the intensity of rehabilitation exercises, the frequency with which they follow the practitioner’s instructions and advice, and their receptivity to changes in the rehabilitation programme during that day’s appointment [42].

#### Interventions used to promote adherence

The reviews included a wide range of different interventions, which we grouped into three different intervention types:

1. *Information provision* and *patient education* were investigated in seven reviews [12, 13, 30, 31, 33, 34, 36], including (i) video- and audio-assisted patient education, (ii) phone calls, (iii) use of supporting materials and spoken or graphically presented information or (iv) other didactical interventions. Patient education has been defined as ‘any combination of learning experiences designed to facilitate voluntary adoption of behaviour conducive to health’ [43]. Niedermann et al. [31] distinguished between ‘purely’ educational programs based on knowledge transfer and psychoeducational programs. In the latter, motivational techniques and shared knowledge-building processes are added to the educational programme, which is done similarly in health coaching [34], and thus also relate to the cognitive, behavioural and relational/psychosocial interventions.
2. *Cognitive and behavioural motivational interventions* were relating frequently to cognitive-behavioural and social-cognitive theories, and applied (i) behavioural graded exercise; (ii) booster sessions, refresher or follow-up in situ by the therapist or via phone call; (iii) behavioural counselling (focusing on readiness to change); (iv) psychoeducational counselling; (v) supervision; (vi) (unspecified) motivational intervention; (vii) positive reinforcement; (viii) action and coping planning; and (ix) goal setting [7, 12, 13, 16, 27, 28, 30, 32–34, 39].
3. *Relational and psychosocial interventions* were less investigated overall. Related aspects included (i) social support; (ii) patient-centeredness, in particular patient-led goal setting, motivational interviewing and the therapeutic or working alliance; and (iii) emotional components [6, 13, 17, 33].

The included reviews focused either on one particular or several types of intervention. Particularly, four reviews [6, 7, 17, 40], which used a BCT taxonomy to analyse the interventions of the primary studies, described BCTs relating to all three intervention types. While this distinction of different types of interventions is useful to showcase the range of diverse interventions and techniques, they do have a great overlap and include a mix of different BCTs. For example, the way of facilitation of information, supervision or goal setting was approached differently according to the relational approach, i.e. being more instructive, directive or more collaborative, participatory, patient-led ([31],cf. [34]).

#### Theoretical underpinning of interventions

No review focused on only one theoretical foundation or excluded studies based on any theoretical model or not underpinning the intervention. In total, the reviews included studies with diverse theoretical models and varying degrees of theoretical underpinning. References to the cognitive behavioural theory (CBT) and to the social-cognitive theory were frequent in the individual studies. Furthermore, the self-determination theory, the transtheoretical model, the health belief model, the social learning theory and the socioemotional selectivity theory were used in some individual studies (cf. [11]). The heterogeneity in the theoretical underpinning of the interventions is reinforced by the given overlap of the theories and models (cf. [11],[28]) and various BCTs are key components of several theories [17]. Furthermore, theories were not used enough to explicitly inform and underpin interventions and they were translated into practise in different ways; thus, interventions based on the same theory may differ substantially [17].

The BCT Taxonomy v1 [8], which relates to various theoretical models, was used in four reviews [6, 7, 17, 40] to identify BCTs in interventions in a standardized manner. The Behaviour Change Wheel [44], which is linked to the BCT Taxonomy v1, was referred to in one review [40] pointing to its usefulness for designing a behaviour change intervention. The number of BCTs used appears to be relevant, as interventions using a higher number (≥ 8) of BCTs achieved a significant effect (pooled SMD = 0.29, 95% CI 0.19–0.40, *p* < 0.001), whereas interventions using a lower number (< 8) of BCTs did not (pooled SMD = 0.08, 95% CI -0.11 to 0.27, *p* = 0.41).

#### Overall efficacy and heterogeneity according to the panoramic meta-analysis

**Fig. 2:**
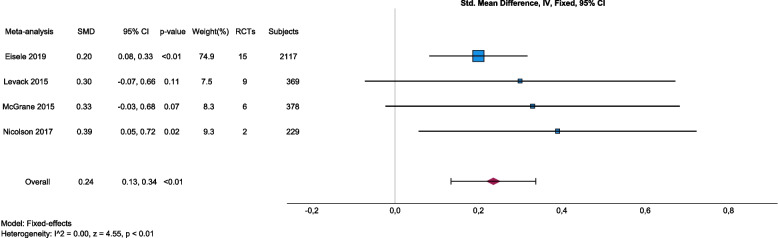
Forest plot of panoramic meta-analysis: interventions aiming at improving adherence, adherence-related outcomes Legend: Eisele 2019. Intervention: Interventions aiming at improving physical activity levels or adherence, containing at least one BCT. Comparison: Usual care, minimal intervention, placebo or no intervention. Outcome: Any measure of physical activity level or adherence to any kind of physical activity. Levack 2015. Intervention: Goal setting (with or without strategies to enhance goal pursuit). Comparison: No goal setting. Outcome: Engagement in rehabilitation. McGrane 2015. Intervention: Motivational interventions as part of a package, psychological strategies, theory-based instructional manuals, Internet-based behavioural programmes and relapse prevention, and re-inforcement strategies. Comparison: Any comparison (not specified). Outcome: Attendance at physiotherapy sessions/exercise classes. Nicolson 2017. Intervention: Booster sessions to increase adherence to therapeutic exercise. Comparison: Contextually equivalent control treatments. Outcome: Self-rated adherence

**Fig. 3:**
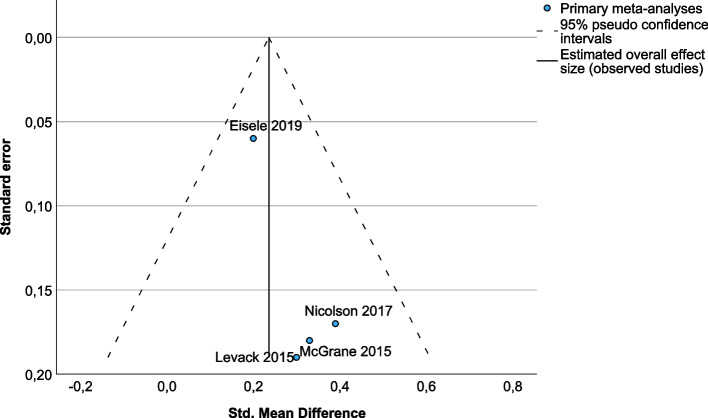
Funnel plot of publication bias

Although there was statistical heterogeneity (*I*^2^ from 41 to 63%) between the primary studies included in each meta-analysis [7, 16, 27, 28], there was no heterogeneity between the pooled effects of these four meta-analyses (*I*^2^ 0%). This means that all variability in the effect size estimates (SMD from 0.20 to 0.39) was attributable to sampling error, but there was no variability in the true effects. Although the interventions were selected based on different eligibility criteria (BCTs, goal-setting strategies, motivational interventions and booster sessions), they appear to be very similar in terms of the effects they trigger. There was no overlap between the primary trials included in the meta-analyses. The pooled SMD was 0.24 (95% CI 0.13, 0.34) (Fig. 2). Effect size estimates were somewhat larger in those meta-analyses with less weight in the model (i.e. due to a larger standard error). However, no obvious publication bias could be detected in the funnel plot (Fig. 3). Sensitivity analyses in the meta-analysis in Eisele et al. [7], considering only studies with PEDro scores of 6 or more, revealed slightly lower effect sizes but still statistically significant effect sizes regarding medium-term effects (SMD_PEDro>=6_ 0.16, 95% CI 0.04–0.28, *p* < 0.01 versus SMD_all_ 0.20, 95% CI 0.08–0.33, *p* < 0.01) and higher numbers of BCTs (SMD_PEDro>=6_ = 0.26, 95% CI 0.16–0.37, *p* < 0.001 versus SMD_all_ = 0.29, 95% CI 0.19–0.40, *p* < 0.001), indicating that low-quality studies may tend to overestimate the efficacy ([7],cf. [31]).

#### Efficacy of informational and educational interventions

The results of five—partly overlapping—reviews [12, 30, 31, 34, 36] showed, with a very low evidence base, that interventions that primarily aimed at information provision and knowledge transfer to the patient had limited efficacy on adherence-related outcomes. There was conflicting evidence and inconsistent efficacy of video-assisted patient education [36] and individualised exercise videos [12, 30] in modifying behaviour or adherence. However, the authors identified the format in which the educational information is presented and the complexity of the addressed behaviour as crucial factors [36]. Videos that provide only spoken or graphically presented health information are inappropriate tools for changing patient behaviour. However, videos with a narrative format appear to be a powerful education tool [36]. Low evidence based on one study [12, 30] indicates that additional written information seems superior to verbal instructions alone (mean difference between groups 39.3%, *p* < 0.001). With a high overlap of studies, two reviews [30, 31] showed that there is limited evidence for long-term effects of patient education targeting knowledge acquisition. While the informative and instructive educational approach is an essential part of patient education, patient education often involves more than the transfer of knowledge [30, 31, 34]. Niedermann et al. [31] compared educational and psychoeducational interventions and provided arguments in favour of psychoeducational approaches that enrich patient education with motivational strategies and techniques (cf. [34]).

#### Efficacy of cognitive and behavioural motivational interventions

Several (though partly overlapping) reviews [12, 16, 28, 30, 33, 37] examined studies on *additional motivational interventions* that were based on social-cognitive or cognitive-behavioural theories. McGrane et al. [28] concluded heterogeneity of motivational interventions, outcomes and measurements as potential causes for conflicting evidence regarding effects on exercise attendance and PT adherence, as they found no significant difference (*p* = 0.07) in exercise attendance between additional motivational intervention groups and their controls (pooled SMD 0.33, 95% CI -0.03 to 0.68, *I*^2^ 62%), but a significant (*p* < 0.01) medium-sized effect of additional motivational interventions on self-efficacy beliefs (pooled SMD 0.71, 95% CI 0.55 to 0.87, *I*^2^ 41%). The heterogeneity hindered in this meta-analysis the statistical analysis of subgroups to determine and compare the efficacy of different components and approaches to motivational interventions [28]. Another meta-analysis [16] found moderate-quality evidence that booster sessions with a physiotherapist helped people with hip/knee osteoarthritis to better adhere to therapeutic exercise (pooled SMD 0.39, 95% CI 0.05 to 0.72, *p* = 0.02, *I*^2^ 35%). Moderate evidence for the efficacy of supervision (2 studies, *n* = 193) favouring adherence was shown [13, 33, 35].

In four reviews [16, 32, 33, 35], four unique high-quality trials supported the use of motivational strategies and behavioural graded exercise to improve adherence to exercise (effect sizes 0.26–1.23)[16]. Behavioural graded exercise includes a preset gradual increase of the physical activity through facility-based interventions followed by booster sessions [45] and uses principles of operant conditioning and self-regulation [16].

While cognitive behavioural programmes seem superior to exercise alone for short-term adherence and clinical attendance [30], behavioural counselling focusing on readiness to change, action and coping plans and/or audio/video exercise cues seem not to improve adherence significantly [16]. Holden [34] concludes inconsistent evidence for health coaching based on the transtheoretical model of change, with one RCT showing some efficacy on exercise compliance (SMD = 1.3). However, the frequently referred to study of Göhner and Schlicht [46], who analysed a cognitive-behavioural intervention with a strong emphasis on action and coping planning [12], showed no difference between experimental and control groups in the first 11 weeks, but a significant difference 5 months later on behaviour (SMD = 0.83) as well as differences over all time-points on self-efficacy (interaction effect of time by group, *F*(3, 43) 10.36, *p* < 0.001, *n* = 47) favouring the intervention [46]. Motivational interventions, including positive reinforcement, increased (i) adherence to home exercise in one RCT [33], (ii) reported frequency of exercise in two RCTs [35] and (iii) self-efficacy beliefs in two RCTs, in the short-term (SMD = 1.23) and in the long-term (SMD = 0.44) ([16],cf. [30]). Self-efficacy beliefs relate to the trust in one’s capacities/competencies to cope with daily demands [47] and are associated (moderate evidence) with adherence [13, 48].

Levack et al. [27] conclude some evidence that goal planning/setting improves engagement in rehabilitation (motivation, involvement and adherence) over the duration of the programme (9 studies, 369 participants, SMD 0.30, 95% CI -0.07 to 0.66). Furthermore, they show a low-quality evidence for effects on patient self-efficacy from more structured goal setting compared to usual care with or without goal setting (2 studies, 134 participants; SMD 0.37, 95% CI 0.02 to 0.71) and from goal setting compared to no goal setting (3 studies; 108 participants; SMD 1.07, 95% CI 0.64 to 1.49). The review did not detect differences in efficacy between the approach taken to goal planning. However and similar to patient education [34], the review authors argue that the lack of clarity about the effects and the low evidence is due to the heterogeneity of the implementation of goal planning, lack of detailed descriptions of the goal-setting process in the intervention groups but also in the control groups, and methodological flaws ([27, 39],cf. [13]).

The BCTs from the cluster *goals and planning* showed various positive effects, although not fully consistently [6, 7, 40]. Eisele et al. [7] identified *goal setting (behaviour)*, *problem-solving*, *goal setting (outcome)*, *action planning* and *reviewing behaviour goal(s)* as often used in non-effective interventions but also in effective ones. A trial that showed negative effects included *problem-solving* and *goal setting (outcome)* as well. Room et al. [40] found one study on older people and Thacker et al. [6] two home-exercise-related studies that used BCTs from the *goals and planning cluster* (i.e. problem-solving and action planning), but none of the studies found differences in favour of the intervention. Willett et al. [17] adjusted the BCTv1 taxonomy to differentiate patient-led and therapist-led goal setting and showed that patient-led goal setting (behaviour) achieved among the highest efficacy ratios across time points.

#### Efficacy of relational and psychosocial interventions

The BCT *Social Support (unspecified)* refers to ‘advise on, arrange or provide social support *(e.g. from friends, relatives, colleagues, ’buddies’ or staff)* or non-contingent praise or reward for the performance of the behaviour*.* It includes encouragement and counselling, but only when it is directed at the behaviour’ [8, Supplementary Material]. Eisele et al. [7] identified this BCT in 19 interventions and 10 control conditions. They found this BCT in three trials supporting efficacy and in seven trials supporting inefficacy. In contrast, Thacker et al. [6] found this BCT in all effective interventions but not in the non-effective ones. Willet et al. [17] concluded from their review that this BCT has among the highest efficacy ratios across time points to promote adherence to physical activity.

Social support may come along with *monitoring and feedback,* which can be graphically or narratively presented by the therapist. Willett et al. [17] recommend that self-monitoring (e.g. activity diaries), feedback on behaviour as well as social support should be used—beyond monitoring purposes—for explicit intervention purposes (e.g. to foster self-efficacy beliefs). *Feedback on behaviour* alone does not seem to be efficacious [6], but feedback can be efficacious for instance in combination with social support or goal setting and planning [17, 40].

Patient-centred approaches were also included in the relational/psychosocial intervention type. Motivational interviewing, which is a collaborative, patient-centred communication style to promote behaviour change [49], was used in three studies, indicating positive effects on exercise compliance, physical activity and exercise at home in two trials, whereas no effect in a pilot study [28]. There is low evidence from three RCTs for positive effects of the therapist-patient alliance on global assessments; however, the efficacy on adherence-related outcomes is unclear [36]. The terms working or therapeutic alliance refer to the social connection or bond between therapist and patient/client, including reciprocal positive feelings, (assertive) communication, empathy, and mutual respect as well as collaboration, shared decision-making, agreement on the treatment goals and tasks [36, 50]. The therapeutic alliance is a patient-centred approach as well. Patient-led goal setting was more often a component within efficacious interventions than therapist-led goal setting [17].

None of the included reviews focused specifically on affective interventions. However, some interventions relate to affective components, for example patient-led goal setting or motivational interviewing may cover emotional needs [27]; health coaching, therapeutic alliance or social support may include emotional support [13, 34, 35, 38]; monitoring may consider emotional consequences [6]; or messaging and information provision may include emotional components [36]. Room et al. [40] included one RCT [51], comparing emotionally meaningful messages against factual informational messages, but with no significant differences between the groups.

#### Efficacy according to the theoretical underpinning

McGrane et al. [28] provide a narrative analysis of the efficacy of interventions according to the different theoretical underpinnings. In their review, the *cognitive-behavioural theory* (CBT) was the most popular theory (4 primary studies) and showed to be efficacious in improving self-efficacy and activity limitations, but not consistently regarding attendance and attrition [28]. The social-cognitive theory was used in three studies, showing improvements in self-efficacy, action and coping planning, and attendance, but conflicting results for exercising in the short and long term. One intervention [52] based on *self-determination theory* showed to be efficacious to improve adherence to physical activity. In contrast to McGrane et al. [28], the reviews [12, 30, 35] point to moderate to conflicting evidence for no or inconsistent efficacy of CBT-based approaches to physiotherapy programmes (see *Efficacy of cognitive and behavioural motivational interventions*). Jordan [35] concluded that the addition of transtheoretical model-based counselling to physiotherapy is no more effective than physiotherapy and a sham intervention (GRADE: High (high quality); Silver). Notably, the interventions may not be representative of the theory described due to diverse translations of the theory into practice and the overlap of the same BCTs among the theories.

Various theories (e.g. the transtheoretical model or the Health Action Process Approach [53]) and studies [54] distinguish the action or adoption phase from the maintenance phase at 6 months. Interestingly, Willet et al. [17] found in total higher short (< 3 months) and long-term (12 months and more) than medium-term (around 6 months) efficacy ratios, pointing to the risk of drop-out when changing from the (short-term) adoption phase to the (long-term) maintenance phase [17]. Eisele et al. [7] divided in their meta-analysis the short-term (< 3 months), medium-term (3–6 months) and long-term (7–12 months post-intervention) differently, showing a small medium-term overall effect (pooled SMD 0.20, 95% CI 0.08–0.33, *p* < 0.01), but no significant long-term effect of interventions comprising BCTs in enhancing physical activity adherence (pooled SMD 0.13, 95% CI 0.02–0.28, *p* = 0.09).

#### Efficacy according to the different types of exercise, physiotherapeutic settings and medical condition

In their Cochrane review, Jordan et al. [35] compared the evidence for the efficacy of different types of exercises and physiotherapy settings. Graded exercise is beneficial for adherence (moderate evidence). The exercise type does not appear to play an important role (moderate evidence). Whether water-based exercise favours adherence is unclear (low evidence and inconsistent results). Furthermore, the supervision of exercising (moderate evidence) is beneficial for adherence, but also self-management programmes improve exercise frequency compared to waiting list or no-intervention control groups (moderate evidence). Exercising individually seems to improve attendance at exercise classes more than exercising in a group (moderate evidence), as individual sessions could be scheduled at more convenient times and missed sessions could be rescheduled, whereas group sessions were scheduled at relatively inflexible times, and missed sessions could not be rescheduled [35]. However, adding group exercise to a home exercise programme can increase overall physical activity levels (moderate evidence) [35]. While the results of home- versus clinic-based interventions were conflicting and confounded by the intervention approaches, a combination of home- and clinic-based approaches may be promising [12] and aligns with the moderate-quality evidence that self-management programmes, refresher or booster sessions with a physiotherapist assist people to better adhere to therapeutic exercise [16].

No study was identified in the reviews that compared other settings, such as private- and public-funded physiotherapy or primary care and rehabilitation settings regarding adherence outcomes. No review and no study comparing the same educational, motivational, or BCT-based intervention across different conditions were identified.

## Discussion

This overview of systematic reviews addresses adherence in the physiotherapy and therapeutic exercise domain, aiming to summarise the evidence on the efficacy of interventions, to explore heterogeneity and to identify research gaps. The overview of reviews provided an adequate approach to generate answers to the research questions. Nineteen reviews, covering 205 unique trials, were included and narratively synthesised. In addition, four meta-analyses were pooled in a panoramic meta-analysis. The findings provide an overview of the diverse interventions and techniques aiming to enhance adherence, ranging from informational/educational to cognitive/behavioural/motivational and to relational/psychosocial intervention types. Furthermore, it synthesised their efficacy in physiotherapy for adults.

Confidence in the reviews was rated moderate or high in four reviews [7, 16, 27, 35], but low or very low in the others (Table 3). The individual reviews considered the evidence levels as mostly low or very low (Table 4; see *Risk of bias and evidence assessment*). Table 5 summarizes the evidence on the efficacy of each intervention and technique according to (a) whether the evidence supports efficacy, (b) the evidence level based on the report in the systematic reviews and (c) the confidence in the reviews as assessed with AMSTAR-2. It must be noted that the components of the intervention which caused the efficacy were not always clear. Some interventions lacked detailed definitions and descriptions of the specific BCTs included [33]. A single technique or mechanism of action was not always identifiable; moreover, various techniques seem to influence each other in such a way that they achieved efficacy only jointly [17, 40].

**Table 5:** Overview of current evidence on the efficacy of interventions and BCTs on adherence-related outcomes

**Intervention/techniques**	**Efficacy supported by the evidence** as reported in the SR	**Level of evidence** as reported in SR	**Confidence in the SR** as assessed with AMSTAR 2	**Source** Review
**Promising interventions/techniques with moderate evidence supporting efficacy**				
Booster session/refresher	Yes	Moderate	**H**, M, L, CL	[35, 40,16^a^,^32^^a^]
Supervision	Yes	Moderate	**H,** L, CL	[35, 33 , 13^a^]
(behavioural) graded activity/exercise	Yes	Moderate	**H**, M, L	[35, 16^a^, 32^a^]
Use of BCTs	Yes	Moderate	**M/H**, L, L	[6, 7, 17]
More BCTs	Yes	Moderate	**M/H**	[7]
Self-management programmes	Yes	Moderate	**H**	[35]
**Possibly promising interventions/techniques with low/very low evidence supporting efficacy**				
Motivational intervention, including positive reinforcement	Yes	Low	**H,** M, CL, CL	[16, 30, 33, 35]
General behaviour change programme	Yes	Low	**CL**, CL	[33, 37]
Cognitive behavioural intervention with coping and action planning	Yes	Low	**CL**	[12, 30]
Goal setting and planning	Yes/inconsistent	Low	**H**, L, L, L, CL	[17, 27,6 13 ,39^a^]
Social support	Yes/inconsistent	Low	**H**, **L**, L	[6, 7, 40]
Counselling	YES/inconsistent	Low	**L**	[13]
Phone surveillance/follow-up	Yes	Low/very low	**L**, CL**,** CL	[13, 32, 33]
Additional written information	Yes	Very low	**CL**, CL	[12, 30^a^
Patient-led goal setting and planning	Yes	Very low	**L**	[17]
Motivational interviewing	Yes	Very low	**L**	[28]
**Interventions/techniques with conflicting evidence (inconsistently) supporting efficacy**				
Feedback and monitoring (alone^b^)	Inconsistent	Low	**L**, L, L	[6, 17, 40]
Health coaching (based on transtheoretical model of change)	Inconsistent	Very low	**L**	[34]
Educational programmes/knowledge provision	Inconsistent	Low	**L**, CL, CL	[31, 36, 40]
Video-assisted education/additional instructions in audio and video format	Inconsistent	Very low/conflicting	**L**, CL, CL, CL	[36, 40,12^a^, 30^a^]
CBT-based approaches to PT programmes	No/inconsistent	Moderate/conflicting	**H** + CL + CL	[12, 30, 35]
**Non-efficacy supported by evidence**				
Behavioural counselling based on transtheoretical model/readiness to change	No	Moderate/low	**H** + M	[16, 35]

Legend: ^a^Indicates that the evidence is based on the same study or studies due to the overlap of the marked reviews, e.g. an overlap of 12 and 15 is marked 12^a^ and 15^a^

^b^The corresponding BCT showed inconsistent efficacy (low evidence) when analysed isolated (alone) but promising efficacy (low/moderate evidence) as one of several BCTs

*CL*, critically low; *L*, low; *M*, moderate; *H*, high confidence in the review

The *efficacy supported by the evidence* is based on the results reported in the reviews. *Yes* refers to results of merely positive effects; meanwhile, *inconsistent* refers to findings of positive and no effects of the intervention (techniques) analysed. *No effects* indicates that the intervention was not efficacious. Negative effects were not reported for the intervention (techniques) shown

The *levels of evidence* were extracted as reported in the reviews, which are based on different evidence appraisal schemes: GRADE (high, moderate, low, very low certainty of evidence), Cochrane Collaboration Back Review Group Evidence Levels (strong, moderate, conflicting, limited, no evidence) and self-developed tools. The levels of evidence were compared across the relevant reviews, considering the confidence in the review and the comprehensiveness of the review

The *confidence in the SR* was assessed with AMSTAR-2 (see Table 4) and presented in the same order as the source review

The interventions (techniques) were ranked according to the displayed three aspects, being on the top of those interventions (techniques) that showed to be efficacious, based on the best available evidence and analysed in reviews with less risk of bias

No clear conclusion can be drawn on the efficacy of informational/educational interventions. Five reviews [12, 30, 31, 34, 36] showed low evidence for the efficacy of interventions on knowledge acquisition and low evidence for limited short-term efficacy on adherence. Providing knowledge alone seems not enough and should be complemented with supportive material (very low evidence) and combined with other interventions (low evidence). Patient education should also include social-cognitive or cognitive-behavioural approaches, psychoeducational interventions and collaborative processes as it is included in the therapeutic alliance approach [31, 34, 36]. Patient education with a more constructive educational approach builds upon the knowledge of the patient, supporting him/her in exploring and co-constructing knowledge which is very relevant in physiotherapy as research has shown [55, 56].

The reviews on additional motivational, cognitive and behavioural interventions showed findings ranging from non-efficacy of behavioural counselling based on readiness to change (with low to moderate evidence) to moderate efficacy for booster sessions and behavioural graded physical activity (with moderate evidence) (see Table 5). Overall, a small overall effect size (SMD 0.24) for motivational interventions is indicative of the findings of the panoramic meta-analysis. The four pooled meta-analyses [7, 16, 27, 28] included studies analysing interventions with a considerable amount of content overlap (e.g. goal-setting and booster sessions are BCTs and often part of motivational interventions), and no statistical heterogeneity of the true effect was found. Nevertheless, the diversity of interventions and techniques included constrain the explanatory power for potential components responsible for the efficacy of adherence. The sensitivity analyses in the meta-analysis of Eisele et al. [7] indicate that low-quality studies tend to overestimate the efficacy (cf. [31]). While some evidence exists on short- and medium-term effects of motivational programmes on adherence, no clear evidence for long-term effects can be concluded [7, 30]. Furthermore, there is moderate and low evidence that additional motivational interventions and goal planning/setting improve adherence to self-efficacy beliefs [27, 28, 39]. Since self-efficacy beliefs play an important role in motivation and adherence [13, 48], the results are relevant for physiotherapists to promote motivation and adherence. Experiencing that one can reach the set goals and manage daily challenges, complemented with feedback and reinforcement from the therapist (or important others), may increase self-efficacy beliefs and human agency [48, 57–59].

A closer look at how and in which manner goals and actions are planned and reviewed seems crucial. The patient-led approach was only reported in 5 of the 26 interventions that incorporated the BCT *goal setting (behaviour)*, although it is associated with greater engagement and achievement than goals which are set by the therapist [17]. Goal setting and action planning should be informed by the patient’s motives, interests and values in order to promote intrinsic motivation, self-determination and subsequently better adherence ([17],cf. [27, 28, 60, 61]). The reviews on the BCTs displayed various positive effects relating to the BCT cluster *goals and planning*; however, they point out that the BCT goal setting is not used alone but in connection with several other BCTs. *Feedback on outcomes of behaviour*, *behavioural contract* and *non-specific reward* as well as *patient led-goal setting*, *self-monitoring of behaviour* and *social support (unspecified)* was included in efficacious interventions [17]. Social support seems to have an important influence on adherence [6, 7, 17, 40], for example through regular phone-calls or home visits, encouraging messaging, supervision or community-based group programs (cf. [1–3],[37, 62]). Social support also relates to the promotion of self-efficacy beliefs, if it endorses confidence in own abilities and competences [6].

Some BCTs seem inherent to standard practices of physiotherapy [6] even though physiotherapists seem to use rather a small number of BCTs [15]. Control groups also contained BCTs [6, 7]; in particular *instruction on how to perform a behaviour*, *generalisation of the target behaviour* and *social support (unspecified)* were frequently coded [6]. Thus, it seems difficult to identify those BCTs that are (most) efficacious in promoting adherence ([7],cf. [50]). Unsurprisingly, the reviews revealed conflicting results and a high risk of bias in the individual studies. However, combining a greater number of BCTs (≥ 8) can be highly recommended, as this achieved a larger effect than interventions using fewer BCTs [7]. It is fairly unlikely that any single BCT changes adherence [6, 7, 17, 40]. In that regard, Ariie et al. [63] argue that not only the amount of BCTs but also the quality, appropriateness and feasibility of the use of the BCTs is crucial.

Meaningful combinations of several BCTs are required. However, the combinations of BCTs may also differ among conditions, personal factors and therapeutic interventions ([7],cf. [63, 64], [64–66]), and over the time. Two reviews consistently point to the same crucial time point (i.e. after 6 months) when BCT efficacy seems to drop, and more attention is required to maintain adherence [7, 17]. *Action planning*, *feedback on behaviour* and *behavioural practice/rehearsal* seem efficacious particularly on short-term. *Patient led-goal setting*, self-monitoring of behaviour and *social support (unspecified)* are among those BCTs that seem more efficacious at long-term [17]. These findings are also in line with findings in non-clinical adults [54] and with motivational theories (e.g. the Health Action Process Approach [53]).

### Limitations

Conducting an overview of reviews is per se associated with methodological limitations. A limitation is that reviews were analysed and not the original RCTs, which adds further risks of bias domains such as selection, analysis and reporting bias. A specific potential source of bias in overviews of reviews is the overlap of primary studies among the included reviews. The small overlap, caused by a few reviews with similar thematic scope, was controlled for in the data analysis. The substantial non-overlap of primary studies across the reviews reflects the clinical and methodological diversity of the included reviews and showcases the efforts to address (a) motivation and (non-)adherence as complex phenomena and from various perspectives.

Another methodological limitation originates from the search strategies. Considering different health-care systems and delimitations of the physiotherapy profession among countries, divergences among the definitions of terms and the use of diverse approaches to physical therapy, physiotherapy or the therapeutic use of exercise and physical activity, made a clear delimitation in the search strategy and inclusion/exclusion criteria difficult. Therefore, we may have missed out some relevant reviews by reducing our search to the two terms physiotherapy and physical therapy. Equally, we may also have included some aspects that were not primarily investigated for physiotherapists or physical therapists. Including only studies with adults, the findings may not be applicable to promote adherence among children.

While we did not exclude reviews from another language, the search was conducted only in English, which may omit important reviews in other languages. All included reviews (and as far as reported, also the original RCTs) were conducted in economically developed countries; however, social-cultural and context-specific factors influence participation and adherence [67–71]. Furthermore, we are aware that our own cultural background and experiences may have influenced the analysis and synthesis of the results and that conclusions drawn in this overview of reviews may not be suitable for every setting around the world. Therefore, we encourage the readers to critically assess the applicability of the findings to their specific context.

Another gap in coverage of this overview is that interventions that were analysed in RCTs but not included in any systematic review are not considered in this overview. Thus, there may be new or alternative intervention approaches that resulted efficacious but were not covered by this overview. Furthermore, reviews that focused only on the use of digital apps or tools, e.g. virtual reality, gamification, exergames or tele-rehabilitation, were excluded from this overview. Several reviews in this field include adherence-related outcomes, showing potential efficacy as well as limitations of the use of digital tools [72–83].

### Research gaps, recommendations and measuring adherence

This overview of reviews highlighted some gaps in the existing knowledge. First, there is a lack of clear evidence on the efficacy of the interventions. The use of BCTs in the intervention as well as in the control groups may be a reason for inconsistent findings and conflicting evidence. Furthermore, the clinical and methodological heterogeneity constrains drawing clear conclusions on the efficacy. Second (and related to the previous), interventions are insufficiently described regarding their theoretical underpinning and active ingredients/techniques and thus limit the comparison of interventions. Theoretical underpinnings were used partly and translated into practise differently. Difficulties concerning the derivation or deduction of concrete, practical techniques or strategies from the theories were reported. A broader use of the BCT taxonomies would make interventions more comparable. Recently, the BCT Ontology was published, which claims to provide a standard terminology and a comprehensive classification system for the content of behaviour change interventions, suitable for describing interventions [84]. Third, there is a need for studies on holistic approaches, complex interventions based on integrative theories and the combination of multiple BCTs. While many theories are based on cognitive and behavioural approaches, affective and psychosocial factors are hardly investigated, overlooked and probably underestimated. Rhodes and Fiala [12] call for studying the influences of affective attitudes on adherence (e.g. enjoyment and pleasing behaviour) which may oppose the more cognitive, instrumental attitudes (e.g. the utility of behaviour). Jordan et al. [35] refer to a meta-analysis in another therapeutic regime [85] to explicit the potential efficacy of affective interventions (e.g. appealing to feelings, emotions or social relationships and social supports) in combination with educational and behavioural interventions on patient adherence [35]. Fourth, more research in patient-led approaches to goal setting and action planning and the relationship of patient-centeredness to adherence is promising [60, 61, 86, 87].

Fifth, the reviews reported many studies that failed to provide evidence for intervention efficacy on adherence, particularly on long-term adherence. There is a need for prolonged observation to investigate long-term effects on adherence. Probably, intervention or follow-up interventions (e.g. booster sessions) must also be prolonged or repeated to avoid drop out to medium-term follow-ups (around 6 months) and to maintain participation. Sixth, studies should pay more attention to the actual efficacy of adherent behaviour on the desired therapeutic outcomes.

Seventh, another research gap lies in the analysis of the potential variation of the intervention efficacy across medical conditions, physiotherapeutic settings, personal characteristics (e.g. age, gender, sociocultural background) and dispositions (e.g. motives, affective attitudes, previous behaviour) and diverse context-related factors. Huynh et al. [79] showed for the case of multiple sclerosis that the efficacy of BCTs is not investigated in all disease stages or throughout the disease course; participants with mild-to-moderate level disability were more frequently included in the studies (cf. [18]). Ariie et al. [73] stated that the response to BCTs may be different according to the condition (cf. [76]). On the one hand, studies analysing the use of the same intervention or same combination of BCTs in different intervention groups (according to the categories mentioned above) could be beneficial for comparison purposes. On the other hand, studies should analyse how to find the ‘right’ (ideally, the ‘most efficacious’) adherence promotion intervention for the patient or target group. Qualitative studies may explore adequate combinations of BCTs and contribute to the understanding of complex intervention processes. The findings showcased that different interventions and BCTs may contribute to adherence and that the BCT Taxonomy defines a wide range of techniques, providing the physiotherapists with an overview of which techniques are useable and thus may inspire and support them to develop additional interventions and to enrich their current physiotherapeutic practise. The physiotherapist may use this knowledge to tailor interventions in a patient-centred manner to promote adherence, and to adapt to the condition, characteristics, dispositions and context-related factors of the patient. Hence, experimental studies could compare the efficacy of tailored to not-tailored interventions.

Finally, the outcome adherence should be better defined and holistically assessed. The definition of adherence (as the extent to which a person’s behaviour corresponds with treatment goals or plans) and calculation of adherence rates (by reported exercise or attended sessions divided by the recommended or prescribed exercise or sessions) are simplifying a complex phenomenon. The average or the percentages of attended or completed sessions do not picture interruptions, regularity or periods of more and less adherence. Attendance regularity can change over the time and different participation and fluctuation patterns can be identified [88, 89]. For example, an adherence rate of 50% can imply (a) that a person attended regularly every second session throughout the period of observation or (b) that a person attended all sessions of the first half of the observation period and then stopped attending. The underlying reasons and motivational factors may be quite different in these two cases. Besides assessing participation and fluctuation patterns, the three dimensions of the SIRAS scale [42], i.e. frequency, intensity and reciprocity, could be considered for a holistic account of adherence. The findings of this overview emphasized the importance of a patient-led goal setting and planning, which includes a shared decision-making process and the mutual agreement to adhere to the jointly established plan (cf. WHO definition of adherence, [5]). The measurement of adherence should be able to distinguish a patient-led approach from a therapist-led approach (cf. [17]) and to appraise the extent of a shared decision-making process. In conclusion, a holistic approach to measure adherence in physiotherapy may include measures of the frequency of attendance/exercising (e.g. attended sessions out of the prescribed/recommended sessions), the regularity of participation and fluctuation (e.g. timeline with pauses and interruptions, visualizing more and less adherent periods), the intensity of attendance/exercising (e.g. the number or the increment of exercises and repetitions performed in comparison to the plan), reciprocity and fidelity to the agreed goals and plan (e.g. therapist’s and patient’s subjective appraisal of the degree of accomplishment of the agreed plan) and persistence/perseverance over the time (e.g. measuring volition via questionnaires or rating persistence in participation in spite of the experienced challenges and barriers).

## Conclusions

We conclude that moderate certainty of evidence supports that (i) additional motivational interventions and behaviour change programmes can increase adherence and patients’ self-efficacy beliefs and (ii) interventions applying BCTs increase adherence, particularly when using a greater number of BCTs and combining various BCTs, and particularly on short to medium term. The BCTs’ *patient-led goal setting*, self-monitoring of behaviour and *social support* seem promising to promote maintenance; (iii) graded activities, booster sessions with a physiotherapist and supervision foster adherence.

There is low certainty of evidence that (i) goal setting and planning improves adherence to treatment regimens, particularly if a patient-centred approach is taken; (ii) motivational interventions including various techniques, such as positive reinforcement, social support, monitoring or feedback, can foster adherence; (iii) social support seems to play an important role in promoting adherence; however, evidence is low as this BCT is frequently found in the control group; and (iv) information provision and transfer of knowledge to the patient may improve adherence-related outcomes when combined with motivational techniques, as in psychoeducational programmes. Additional written information is superior to verbal instructions alone; (v) a combination of home-based exercise programmes with clinical supervision, refresher or booster sessions, or/and self-management programmes seems promising to increase adherence.

Regarding the implications for future research, a holistic approach to measure adherence in physiotherapy and the investigation of clearly defined interventions combining multiple BCTs is recommended.

### Supplementary Information

**Additional file 1: **Search details

**Additional file 2: **Overlap of reviews

## Data Availability

All data generated or analysed during this study are included in this published article and its supplementary information files.

## Abbreviations

BCT: Behaviour change technique
CB/CBT: Cognitive behavioural/cognitive behavioural theory
CG: Control/comparator group
GRADE: Grades of Recommendation, Assessment, Development and Evaluation
IG: Intervention/experimental group
PA: Physical activity
PRIOR: Preferred Reporting Items for Overviews of Reviews
PRISMA: Preferred Reporting Items for Systematic Reviews and Meta-Analysis
PT: Physiotherapy
RCT: Randomised controlled trial
SMD: Standardised mean difference
SR: Systematic review

## Footnotes

1 Overview of reviews, umbrella review and reviews of reviews are considered as synonyms in this article (cf. [19]).
