# Supplementary material for: Efficacy of interventions and techniques on adherence to physiotherapy in adults: an overview of systematic reviews and panoramic meta-analysis
Source: Syst Rev. 2024 May 21;13:137. doi: 10.1186/s13643-024-02538-9 (PMC11106864; doi:10.1186/s13643-024-02538-9)
Supplement: Supplementary file 1 — Additional file 1: Search details [file 13643_2024_2538_MOESM1_ESM.pdf]

Additional file 1: search details

All databases were last searched on 31st of January 2023

Database	Keyword combination
Pubmed (National Library of Medicine)	(physiotherap* OR "physical therapy") AND (motivation* OR "behaviour change" OR "behavior change" OR centeredness OR "therapeutic alliance" OR "working alliance" OR "health education" OR communication OR counselling) AND (adheren* OR complian*) AND (effectiveness OR effect*)<br>Filter: only reviews, systematic review and meta-analysis
Web of Science Core Collection (Clarivate Analytics)	TS=(physiotherap* OR "physical therapy") AND TS=(motivation* OR "behaviour change" OR "behavior change" OR centeredness OR "therapeutic alliance" OR "working alliance" OR "health education" OR communication OR counselling) AND TS=(adheren* OR complian*) AND TS=(effectiveness OR effect*) Filter: only review articles
Cochrane Library (Wiley)	(physiotherap* OR "physical therapy") AND (motivation* OR "behaviour change" OR "behavior change" OR centeredness OR "therapeutic alliance" OR "working alliance" OR "health education" OR communication OR counselling) AND (adheren* OR complian*) AND (effectiveness OR effect*) in Title Abstract Keyword
CINAHL complete (EBSCOhost)	(physiotherap* OR "physical therapy") AND (motivation* OR "behaviour change" OR "behavior change" OR centeredness OR "therapeutic alliance" OR "working alliance" OR "health education" OR communication OR counselling) AND (adheren* OR complian*) AND (effectiveness OR effect*) AND ("systematic review" OR meta-analysis)<br>Filter: Academic journals (no dissertations)
PsycInfo (EBSCOhost)	1. (physiotherap* OR "physical therapy") AND (motivation* OR "behaviour change" OR "behavior change") AND (adheren* OR complian*) AND (effectiveness OR effect*). Expanders - Apply equivalent subjects; Search modes - Boolean/Phrase. Filter: Literature Review; SR; Meta-analysis; Meta-synthesis; AND peer-reviewed.<br>2. (physiotherap* OR "physical therapy") AND (centeredness OR "therapeutic alliance" OR "working alliance" OR "health education" OR communication OR counselling) AND (adheren* OR complian*) AND (effectiveness OR effect*). Expanders - Apply equivalent subjects; Search modes - Boolean/Phrase. Filter: Literature Review; SR; Meta-analysis; Meta-synthesis; AND peer-reviewed
Scopus (Elsevier)	1. TITLE-ABS-KEY ((physiotherap* OR "physical therapy") AND (motivation* OR "behaviour change" OR "behavior change") AND (adheren* OR complian*) AND (effectiveness OR effect*)) AND (LIMIT-TO (DOCTYPE , "re")) AND (LIMIT-TO (SRCTYPE , "j"))<br>2. TITLE-ABS-KEY ((physiotherap* OR "physical therapy") AND (centeredness OR "therapeutic alliance" OR "working alliance" OR "health education" OR communication OR counselling) AND (adheren* OR complian*) AND (effectiveness OR effect*)) AND (LIMIT-TO (SRCTYPE , "j")) AND (LIMIT-TO (DOCTYPE , "re"))
PEDro (Physiotherapy Evidence Database)	physiotherap* motivation* adheren* effect*<br>physiotherap* behaviour change adheren* effect* physiotherap* behavior change adheren* effect*<br>physical therap* motivation* adheren* effect*<br>physical therap* behavior change adheren* effect*<br>physiotherap* adheren* effect* centered*<br>physiotherap* adheren* effect* centred*<br>physiotherap* adheren* effect* "therapeutic alliance"<br>physiotherap* adheren* effect* "working alliance"<br>physiotherap* adheren* effect* "health education"<br>physiotherap* adheren* effect* communication<br>physiotherap* adheren* effect* counselling<br>physical therap* adheren* effect* centered*<br>physical therap* adheren* effect* centred*<br>physical therap* adheren* effect* "therapeutic alliance"<br>physical therap* adheren* effect* "working alliance"<br>physical therap* adheren* effect* "health education"<br>physical therap* adheren* effect* communication<br>physical therap* adheren* effect* counselling<br>Filter: systemtic reviews
