# Supplementary material for: Efficacy of interventions and techniques on adherence to physiotherapy in adults: an overview of systematic reviews and panoramic meta-analysis
Source: Syst Rev. 2024 May 21;13:137. doi: 10.1186/s13643-024-02538-9 (PMC11106864; doi:10.1186/s13643-024-02538-9)
Supplement: Supplementary file 2 — Additional file 2: Overlap of reviews [file 13643_2024_2538_MOESM2_ESM.pdf]

Abu Abed, M., W. Himmel, S. Vormfelde, und J. Koschack. „Video-assisted patient education to modify behavior: A systematic review“. Patient Education and Counseling 97, Nr. 1 (2014): 16–22. https://doi.org/10.1016/j.pec.2014.06.015.	Areerak, K., P. Waongenngarm, und P. Janwantanakul. „Factors associated with exercise adherence to prevent or treat neck and low back pain: A systematic review“. Musculoskeletal Science and Practice 52 (2021). https://doi.org/10.1016/j.msksp.2021.102333.	Bachmann, C, P Oesch, und S Bachmann. „Recommendations for Improving Adherence to Home-Based Exercise: A Systematic Review“. PHYSIKALISCHE MEDIZIN REHABILITATIONSMEDIZIN KURORTMEDIZIN 28, Nr. 1 (Januar 2018): 20–31. https://doi.org/10.1055/s-0043-120527.	Beinart, Naomi A., Claire E. Goodchild, John A. Weinman, Salma Ayis, und Emma L. Godfrey. „Individual and Intervention-Related Factors Associated with Adherence to Home Exercise in Chronic Low Back Pain: A Systematic Review“. The Spine Journal 13, Nr. 12 (Dezember 2013): 1940–50. https://doi.org/10.1016/j.spinee.2013.08.027.
Bassett et al. 2010<br>Doering et al. 2001<br>Dyson et al. 2010<br>Gerber BS [10] 2005<br>Hagan et al. 1983<br>Haines et al. 2009<br>Houston et al. 2011<br>Huang et al. 2009<br>Kinnane et al. 2008<br>Mazor et al. 2006<br>McCulloch et al. 1983<br>Mulrow et al. 1986<br>O'Donnell et al. 1997<br>Opat et al. 2000<br>Powell et al. 1995<br>Savage et al. 2003<br>Self et al. 1983<br>Solomon et al. 1988<br>van der Palen et al. 1997<br>Wiese et al. 2005	Basler et al. (2007)<br>Coppack et al. (2012)<br>Hügli et al. (2015)<br>Lonsdale et al. (2017)<br>Gialanella et al. (2020)<br>Andersen et al. (2011)<br>Dalager et al. (2015)<br>Friedrich et al. (1998)<br>Pedersen et al. (2013)	Henry KD et al. (1999)<br>McAuley E et al. (1994)	Donzelli et al. (2006)<br>Friedrich et al. (1998;2005)<br>Harkapaa et al. (1991)<br>Kuukanen et al. (2007)<br>Linton et al. (1996)<br>Ljunggren et al. (1997)<br>Reilly et al. (1989)<br>Soukup et al. (1999;2001)<br>Vong et al. (2011)

Cinthuja P, Krishnamoorthy N, Shivapatham G. Effective interventions to improve long-term physiotherapy exercise adherence among patients with lower limb osteoarthritis. A systematic review. BMC Musculoskelet Disord. 2022;23:147	Eisele, A, D Schagg, LV Kramer, J Bengel, und W Gohner. „Behaviour change techniques applied in interventions to enhance physical activity adherence in patients with chronic musculoskeletal conditions: A systematic review and meta-analysis“. PATIENT EDUCATION AND COUNSELING 102, Nr. 1 (Januar 2019): 25–36. https://doi.org/10.1016/j.pec.2018.09.018.	Hall, Amanda M., Paulo H. Ferreira, Christopher G. Maher, Jane Latimer, und Manuela L. Ferreira. „The Influence of the Therapist-Patient Relationship on Treatment Outcome in Physical Rehabilitation: A Systematic Review“. Physical Therapy 90, Nr. 8 (1. August 2010): 1099–1110. https://doi.org/10.2522/ptj.20090245.	Holden, J., M. Davidson, und P. D. O’Halloran. „Health Coaching for Low Back Pain: A Systematic Review of the Literature“. International Journal of Clinical Practice 68, Nr. 8 (August 2014): 950–62. https://doi.org/10.1111/ijcp.12444.
Benell et al. 2017<br>Baker et al. 2020<br>Brosseau et al. 2012<br>Pisters et al. 2010<br>Hughes, 2010	Åsenlöf et al. (2009)<br>Basler et al. (2007)<br>Ben-Ami et al. (2017)<br>Bennell et al. (2014)<br>Bennell et al. (2017)<br>Brosseau et al. (2012)<br>Cederbom et al. (2014)<br>Ersek et al. (2008)<br>Focht et al. (2014)<br>Hurley et al. (2015)<br>Hutting et al. (2015)<br>Magalhães et al. (2017)<br>McDonough et al. (2013)<br>Meng et al. (2011)<br>Rasmussen-Barr et al. (2009)<br>Ravaud et al. (2009)<br>Schaller et al. (2017)<br>Schlenk et al. (2011)<br>Svege et al. (2015)<br>Teirlinck et al. (2016)<br>Vonk et al. (2009)<br>Yip et al. (2007)	Ferreira et al (2009)<br>Zaproudina et al (2007)<br>Zaproudina et al (2009)	Vong et al. (2011)<br>Basler et al. (2007)<br>Becker et al. (2008) / Leonhardt et<br>Iles et al. (2011)

Jordan, Joanne L., Melanie A. Holden, Elizabeth Ej Mason, und Nadine E. Foster. „Interventions to Improve Adherence to Exercise for Chronic Musculoskeletal Pain in Adults". The Cochrane Database of Systematic Reviews, Nr. 1 (20. Januar 2010): CD005956. https://doi.org/10.1002/14651858.CD005956.pub2.	Levack, William M. M., Kathryn Taylor, Richard J. Siegert, Sarah G. Dean, Kath M. McPherson, und Mark Weatherall. „Is Goal Planning in Rehabilitation Effective? A Systematic Review". Clinical Rehabilitation 20, Nr. 9 (September 2006): 739–55. https://doi.org/10.1177/0269215506070791.	Levack, W.M.M., M. Weatherall, E.J.C. Hay-Smith, S.G. Dean, K. Mcpherson, und R.J. Siegert. „Goal setting and strategies to enhance goal pursuit for adults with acquired disability participating in rehabilitation". Cochrane Database of Systematic Reviews 2015, Nr. 7 (2015). https://doi.org/10.1002/14651858.CD009727.pub2.	McGrane, N, R Galvin, T Cusack, und E Stokes. „Addition of motivational interventions to exercise and traditional Physiotherapy: a review and meta-analysis". PHYSIOTHERAPY 101, Nr. 1 (März 2015): 1–12. https://doi.org/10.1016/j.physio.2014.04.009.
Asenlof 2005<br>Barlow 2000<br>Basler 2007<br>Bernaards 2007/2006<br>Blixen 2004<br>Carr 2005<br>Cohen 1983 / Heinrich<br>Ersek 2004; 2008<br>Ettinger / Messier / Rejeski 1997<br>Ferreira 2007<br>Fransen 2007<br>Friedrich 1996<br>Friedrich 1998<br>Fries 1997<br>Halbert 2001<br>Harkapaa 1990<br>Huang 2003<br>Huang 2005<br>Hughes 2004<br>Hurley 2007<br>Jensen 2001<br>Koumantakis 2005<br>Lorig 1985<br>Luszczynska 2006<br>Mangione 1999<br>McCarthy 2004<br>Mikesky 2006<br>Minor 1989<br>Nour 2006<br>Petrella 2000<br>Schoo 2005<br>Sherman 2005<br>Smeets 2006<br>Soderlund 2001<br>Song 2003<br>Soukup 1999<br>Taimela 2000<br>Talbot 2003<br>Veenhof 2006<br>Viljanen 2003<br>Yip 2007<br>Ylinen 2003	Arnetz et al. (2004)<br>Bassett and Petrie (1999)<br>Bell et al. (2003)<br>Blair et al. (1996)<br>Blair (1995)<br>Cross and Parsons (1971)<br>Duncan and Pozehl (2002, 2003)<br>Gauggel and Billino (2002)<br>Gauggel and Fischer (2001)<br>Gauggel et al. (2001)<br>Gauggel et al. (2002)<br>Hart (1978)<br>Howell (1986)<br>Levine et al. (2000)<br>Mann and Sullivan (1987)<br>Ostelo et al. (2000, 2003, 2004)<br>Scott et al. (2004)<br>Sperduto et al. (1986)<br>Webb and Glueckauf (1994)	Arnetz 2004;<br>Asenlof 2005;<br>Bassett 1999;<br>Bell 2003;<br>Blair 1991<br>Blair 1996;<br>Cheng 2012;<br>Conrad 2000;<br>Coote 2012;<br>Coppack 2012;<br>Cross 1971;<br>Culley 2010;<br>Duncan 2003;<br>Evans 2002;<br>Fredenburgh 1993;<br>Gagné 2003;<br>Hart 1978;<br>Hart 2002;<br>Harwood 2012;<br>Holliday 2007;<br>Howell 1986;<br>Iacovino 1997;<br>James 1993<br>Jonsdottir 2012;<br>LaFerriere 1978;<br>Mann 1987;<br>McPherson 2009;<br>Miller 2012;<br>O'Brien 2013;<br>Oestergaard 2012;<br>Ostelo 2003;<br>Parsons 2012;<br>Richardson 2007;<br>Scott 2004;<br>Sewell 2005;<br>Stanhope 2013;<br>Taylor 2012<br>Webb 1994;<br>Woltmann 2011	Annesi et al. (2011)<br>Asenlof et al. (2005)<br>Basler et al. (2007)<br>Befort et al. (2008)<br>Brodie et al. (2008, 2005)<br>Friedrich et al. (2005, 1998)<br>Göhner and Schlicht (2006)<br>Marcus and Stanton (1993)<br>Millen and Bray (2009)<br>Schelling et al. (2009)<br>Silva et al. (2010)<br>Sniehotta et al. (2005)<br>van Weert et al. (2010)<br>Vong et al. (2011)

McLean, Sionnadh Mairi, Maria Burton, Lesley Bradley, und Chris Littlewood. „Interventions for Enhancing Adherence with Physiotherapy: A Systematic Review“. Manual Therapy 15, Nr. 6 (Dezember 2010): 514–21. https://doi.org/10.1016/j.math.2010.05.012.	Nicolson, Philippa J. A., Kim L. Bennell, Fiona L. Dobson, Ans Van Ginckel, Melanie A. Holden, und Rana S. Hinman. „Interventions to Increase Adherence to Therapeutic Exercise in Older Adults with Low Back Pain and/or Hip/Knee Osteoarthritis: A Systematic Review and Meta-Analysis“. British Journal of Sports Medicine 51, Nr. 10 (Mai 2017): 791–99. https://doi.org/10.1136/bjsports-2016-096458.	Niedermann, K., J. Fransen, R. Knols, und D. Uebelhart. „Gap between short- and long-term effects of patient education in rheumatoid arthritis patients: A systematic review“. Arthritis Care and Research 51, Nr. 3 (2004): 388–98. https://doi.org/10.1002/art.20399.	Rhodes, Ryan E., und Bonnie Fiala. „Building Motivation and Sustainability into the Prescription and Recommendations for Physical Activity and Exercise Therapy: The Evidence“. Physiotherapy Theory and Practice 25, Nr. 5–6 (Juli 2009): 424–41. https://doi.org/10.1080/09593980902835344.
Basler et al. (2007)<br>Friedrich et al. (1998, 2005)<br>Gohner and Schlicht (2006)<br>Lysack et al. (2005)<br>Schneiders et al. (1998)	Basler et al, 2007<br>Bennell et al, 2014 (b)<br>Brosseau et al,2012<br>Freidrich et al,1998<br>O’Brien et al, 2013<br>Pisters et al, 2010<br>Schoo et al, 2005<br>Tuzun et al, 2012<br>Vong et al, 2011	Barlow et al. (1998)<br>Bradley et al. (1987)<br>Brus et al. (1998)<br>Hammond et al. (1999)<br>Helliwell et al. (1999)<br>Kraaimaat et al. (1995)<br>Lindroth et al. (1997)<br>Parker (1988)<br>Parker (1995)<br>Scholten (1999)<br>Taal et al. (1993)	Basler, et al. 2007<br>Bassett and Petrie 1999<br>Bassett and Prapavessis 2007<br>Friedrich, et al. 1998<br>Gohner and Schlicht 2006<br>Schneiders, Zusman, and Singer, 1<br>Schoo, Morris, and Bui 2005

Room, Jonathan, Erin Hannink, Helen Dawes, und Karen Barker. „What Interventions Are Used to Improve Exercise Adherence in Older People and What Behavioural Techniques Are They Based on? A Systematic Review“. BMJ Open 7, Nr. 12 (14. Dezember 2017): e019221. https://doi.org/10.1136/bmjopen-2017-019221.	Thacker, J, F Bosello, und C Ridehalgh. „Do behaviour change techniques increase adherence to home exercises in those with upper extremity musculoskeletal disorders? A systematic review“. MUSCULOSKELETAL CARE, o. J. https://doi.org/10.1002/msc.1532.	Willett, Matthew, Joan Duda, Sally Fenton, Charlotte Gautrey, Carolyn Greig, und Alison Rushton. „Effectiveness of Behaviour Change Techniques in Physiotherapy Interventions to Promote Physical Activity Adherence in Lower Limb Osteoarthritis Patients: A Systematic Review“. Herausgegeben von Jean-Philippe Regnaux. PLOS ONE 14, Nr. 7 (10. Juli 2019): e0219482. https://doi.org/10.1371/journal.pone.0219482.
Boshuizen et al (2005)<br>Cheetham et al (2004)<br>Duncan and Pozehl (2003)<br>Gallagher (2016)<br>Gardner et al (2011)<br>Ridgel et al (2016)<br>Schneider et al (2011)<br>Schoo et al (2005)<br>Steele et al (2008)<br>Wu et al (2010)<br>Yates et al (2005)	Chen et al., 2017;<br>Clark et al., 2019;<br>Gialanella et al., 2020;<br>Ludvigsson et al., 2016<br>Martinez Rico et al., 2018<br>Salo et al., 2012	Bennell 2005<br>Bennell 2010<br>Bennell 2014<br>Bennell 2014b<br>Bennell 2016<br>Bennell 2017<br>Crossley 2015<br>Deyle 2000<br>Dincer 2016<br>EMPART 2013<br>Hiyama 2012<br>Hunt 2010<br>Jones 2012<br>Kawasaki 2009<br>Kuru-Colak 2017<br>Lim 2008<br>MOA 2013<br>Odole 2013<br>Schlenk 2011<br>Segal 2015<br>Teirlinck 2016<br>Van Baar 1998<br>Veenhof 2006<br>Wallis 2017

List of unique trials	Frequency
Basler et al. (2007)	8
Friedrich et al. (1998, 2005)	7
Schoo et al (2005)	4
Vong et al. (2011)	4
Asenlof et al. (2005)	3
Bassett and Petrie (1999)	3
Bennell et al. (2017)	3
Brosseau et al. (2012)	3
Duncan and Pozehl (2002, 2003)	3
Gohner and Schlicht (2006)	3
Arnetz et al. (2004)	2
Bell et al. (2003)	2
Bennell et al. (2014)	2
Bennell 2014b	2
Blair et al. (1996)	2
Coppack et al. (2012)	2
Cross and Parsons (1971)	2
Ersek et al. (2008)	2
Gauggel et al. (2001)	2
Gauggel et al. (2002)	2
Gialanella et al. (2020)	2
Hart (1978)	2
Howell (1986)	2
Mann and Sullivan (1987)	2
O'Brien et al, 2013	2
Ostelo et al. (2000, 2003, 2004)	2
Pisters et al, 2010	2
Schlenk et al. (2011)	2
Schneiders et al. (1998)	2
Scott et al. (2004)	2
Soukup et al. (1999;2001)	2
Teirlinck et al. (2016)	2
Veenhof 2006	2
Webb and Glueckauf (1994)	2
Yip et al. (2007)	2
Andersen et al. (2011)	1
Annesi et al. (2011)	1
Åsenlöf et al. (2009)	1
Baker et al. 2020	1
Barlow 2000	1
Barlow et al. (1998)	1
Bassett and Prapavessis 2007	1
Bassett et al. 2010	1
Becker et al. (2008) / Leonhardt et	1
Befort et al. (2008)	1
Ben-Ami et al. (2017)	1
Bennell 2005	1
Bennell 2010	1
Bennell 2016	1
Bernaards 2007/2006	1
Blair (1991)	1
Blair (1995)	1
Blixen 2004	1
Boshuizen et al (2005)	1
Bradley et al. (1987)	1

Brodie et al. (2008, 2005)	1
Brus et al. (1998)	1
Carr 2005	1
Cederbom et al. (2014)	1
Cheetham et al (2004)	1
Chen et al., 2017;	1
Cheng 2012;	1
Clark et al., 2019;	1
Cohen 1983 / Heinrich	1
Conrad 2000;	1
Coote 2012;	1
Crossley 2015	1
Culley 2010;	1
Dalager et al. (2015)	1
Deyle 2000	1
Dincer 2016	1
Doering et al. 2001	1
Donzelli et al. (2006)	1
Dyson et al. 2010	1
EMPART 2013	1
Ettinger / Messier / Rejeski 1997	1
Evans 2002;	1
Ferreira 2007	1
Ferreira et al (2009)	1
Focht et al. (2014)	1
Fransen 2007	1
Fredenburgh 1993;	1
Friedrich 1996	1
Fries 1997	1
Gagné 2003;	1
Gallagher (2016)	1
Gardner et al (2011)	1
Gerber BS [10] 2005	1
Hagan et al. 1983	1
Haines et al. 2009	1
Halbert 2001	1
Hammond et al. (1999)	1
Harkapaa 1990	1
Harkapaa et al. (1991)	1
Hart 2002;	1
Harwood 2012;	1
Helliwell et al. (1999)	1
Henry KD et al. (1999)	1
Hiyama 2012	1
Holliday 2007;	1
Houston et al. 2011	1
Huang 2003	1
Huang 2005	1
Huang et al. 2009	1
Hughes 2004	1
Hughes, 2010	1
Hügli et al. (2015)	1
Hunt 2010	1
Hurley 2007	1
Hurley et al. (2015)	1
Hutting et al. (2015)	1
Iacovino 1997;	1

Iles et al. (2011)	1
James 1993	1
Jensen 2001	1
Jones 2012	1
Jonsdottir 2012;	1
Kawasaki 2009	1
Kinnane et al. 2008	1
Koumantakis 2005	1
Kraaimaat et al. (1995)	1
Kuru-Colak 2017	1
Kuukanen et al. (2007)	1
LaFerriere 1978;	1
Levine et al. (2000)	1
Lim 2008	1
Lindroth et al. (1997)	1
Linton et al. (1996)	1
Ljunggren et al. (1997)	1
Lonsdale et al. (2017)	1
Lorig 1985	1
Ludvigsson et al., 2016	1
Luszczynska 2006	1
Lysack et al. (2005)	1
Magalhães et al. (2017)	1
Mangione 1999	1
Marcus and Stanton (1993)	1
Martinez Rico et al., 2018	1
Mazor et al. 2006	1
McAuley E et al. (1994)	1
McCarthy 2004	1
McCulloch et al. 1983	1
McDonough et al. (2013)	1
McPherson 2009;	1
Meng et al. (2011)	1
Mikesky 2006	1
Millen and Bray (2009)	1
Miller 2012;	1
Minor 1989	1
MOA 2013	1
Mulrow et al. 1986	1
Nour 2006	1
O'Donnell et al. 1997	1
Odole 2013	1
Oestergaard 2012;	1
Opat et al. 2000	1
Parker (1995)	1
Parker (1988)	1
Parsons 2012;	1
Pedersen et al. (2013)	1
Petrella 2000	1
Powell et al. 1995	1
Rasmussen-Barr et al. (2009)	1
Ravaud et al. (2009)	1
Reilly et al. (1989)	1
Richardson 2007;	1
Ridgel et al (2016)	1
Salo et al., 2012	1
Savage et al. 2003	1

Schaller et al. (2017)	1
Schelling et al. (2009)	1
Schneider et al (2011)	1
Scholten (1999)	1
Segal 2015	1
Self et al. 1983	1
Sewell 2005;	1
Sherman 2005	1
Silva et al. (2010)	1
Smeets 2006	1
Sniehotta et al. (2005)	1
Soderlund 2001	1
Solomon et al. 1988	1
Song 2003	1
Sperduto et al. (1986)	1
Stanhope 2013;	1
Steele et al (2008)	1
Svege et al. (2015)	1
Taal et al. (1993)	1
Taimela 2000	1
Talbot 2003	1
Taylor 2012	1
Tuzun et al, 2012	1
Van Baar 1998	1
van der Palen et al. 1997	1
van Weert et al. (2010)	1
Viljanen 2003	1
Vonk et al. (2009)	1
Wallis 2017	1
Wiese et al. 2005	1
Woltmann 2011	1
Wu et al (2010)	1
Yates et al (2005)	1
Ylinen 2003	1
Zaproudina et al (2007)	1
Zaproudina et al (2009)	1
